# Artificial intelligence in gastric cancer research: a bibliometric and visualized analysis from 1993 to 2026

**DOI:** 10.3389/fonc.2026.1926967

**Published:** 2026-09-14

**Authors:** Xueqing Wang, Changzhu Zhang, Yanchun Ma, Ziliang Liu, Guoying Liang

**Affiliations:** 1 The First School of Clinical Medicine, Heilongjiang University of Chinese Medicine, Harbin, China; 2 Department of Radiology, Qiqihar Hospital of Chinese Medicine, Qiqihar, China; 3 Academic Research Department, Heilongjiang University of Chinese Medicine, Harbin, China; 4 Department of Gastroenterology 1, The First Affiliated Hospital of Heilongjiang University of Chinese Medicine, Harbin, China

**Keywords:** artificial intelligence, gastric cancer, bibliometric, CiteSpace, VOSviewer

## Abstract

**Background:**

Gastric cancer (GC) is the fifth most common cancer worldwide, ranking fifth in both incidence and mortality rates; it severely impacts patients’ quality of life, and the identification and detection of GC are crucial for its prevention. In recent years, there has been a growing trend in the application of artificial intelligence (AI) for the diagnosis, treatment and prognosis of GC; however, systematic analyses using bibliometric tools remain scarce. We have conducted a comprehensive bibliometric analysis to assess the current state and future trends of research on the AI in GC, thereby providing valuable insights to inform further in-depth research in this field.

**Objective:**

To utilize bibliometric and network analysis methods to examine research progress and trends of AI applications in GC. The findings of this study aim to provide a foundation and guidance for further in-depth research into GC.

**Methods:**

A dual-database search and analysis were conducted. Firstly, English-language academic journals in the Web of Science Core Collection (WoSCC) database on the application of AI in GC were retrieved. Subsequently, VOSviewer was utilized to conduct a network co-occurrence analysis of the output data, including institutional affiliations, authors, references and keywords. CiteSpace software was employed to perform statistical analyses of annual publication numbers, keyword clustering, citation counts and keyword bursts. Scimago Graphica was used to map collaboration networks between countries and regions. Finally, we searched PubMed for clinical trial literature to conduct a complementary analysis, which improved the scientific rigor and comprehensiveness of our results.

**Results:**

A total of 2,357 eligible articles and 311 clinical trials were included. Since 1993, the number of published papers has increased steadily each year. Among the authors, several stable core groups have emerged, represented by Li, Wang, Tian, Dong, Ta Da and Yu, among others. Of the affiliated institutions, the Chinese Academy of Sciences showed the strongest association and published the most papers, totaling 102. High-frequency keywords include ‘gastric cancer’, ‘machine learning’, ‘artificial intelligence’, ‘deep learning’ and ‘radiomics’. Results derived from PubMed complement those from the WoSCC dataset, offering a more holistic overview of the research landscape in this field.

**Conclusion:**

The visualized map provides an intuitive overview of research landscape of AI applications in GC over the past 33 years. Over the last five years, research in this field has gradually intensified, and the trend is positive. Our findings indicate that AI-assisted endoscopic and pathological diagnosis of GC, immunotherapy, and AI-assisted clinical decision-making systems will be key areas of future research.

## 1 Introduction

Recent data from the World Health Organisation (WHO) indicate that gastric cancer (GC) ranks fifth worldwide in terms of both incidence and mortality (1).Artificial Intelligence (AI), combined with radiomics, aids in the endoscopic and pathological diagnosis of GC as well as immunotherapy, thereby significantly improving the standard of cancer treatment. In traditional diagnostic approaches, gastroscopy and pathological biopsy are the gold standards for confirming a diagnosis of GC. Researchers have utilized deep learning(DL) techniques to analyze endoscopic, computed tomography (CT) and histopathological images, enabling the diagnosis, staging and prognostic prediction of GC (2). Machine learning(ML) can be applied in early diagnosis, while research into molecular mechanisms and combined laparoscopic and endoscopic surgery have also pointed to future directions for the treatment of GC (3).AI-based image analysis can improve the diagnosis, staging, prognostic prediction and treatment efficacy assessment of GC (4).

Bibliometric analysis has emerged as a powerful tool for evaluating the current research landscape and identifying emerging trends across diverse disciplines, including oncology and gastroenterology (5). This methodology leverages quantitative techniques and well-established algorithms to aggregate extensive bibliometric datasets (6). Researchers can intuitively interpret publication characteristics and research hotspots via such analyses, which further support the evaluation of research output, detection of emerging trends, and evidence-based decision-making. VOSviewer is open-source software capable of processing large-scale datasets to construct co-authorship networks, keyword co-occurrence networks, and citation networks. CiteSpace is developed to visualize patterns and evolutionary tendencies within scientific literature, with a particular focus on temporal co-citation network visualization and the identification of influential research frontiers. In recent years, studies on AI applications in GC have proliferated; nevertheless, a systematic bibliometric analysis focusing on this research area is still lacking (7). In the present study, VOSviewer and CiteSpace were adopted to conduct a bibliometric analysis of publications on AI applications in GC research published from 1993 to 2026. This study aims to characterize the overall research landscape and temporal trends in this field, thereby providing valuable references for researchers in related disciplines.

## 2 Materials and methods

### 2.1 Data sources

Two databases were retrieved for subsequent analysis. Initially, search terms for the Web of Science Core Collection (WoSCC) were compiled by integrating MeSH terms for GC, terms from previous bibliometric studies, and professional terms pertaining to AI. The retrieval strategy adopted was as follows: “TS=((“gastric cancer” OR “stomach cancer” OR “gastric neoplasms” OR “gastric carcinoma” OR “gastric adenocarcinoma” OR “gastric tumor*”) AND (“artificial intelligence” OR AI OR “machine learning” OR “deep learning” OR “neural network*” OR “computer vision” OR “radiomics” OR “predictive analytics”))”. The search was conducted using subject terms with no restriction on the starting publication year, while the cutoff date was set to June 1, 2026. A total of 2,855 publications spanning 1993 to 2026 were initially retrieved. Document types were limited to articles and review articles. Conference abstracts, letters, irrelevant records and non-English publications were excluded. Ultimately, 2,357 eligible publications were included as the dataset for subsequent visual analysis. The literature screening process was illustrated in **Figure 1**. Subsequently, we performed an identical search in PubMed using the following query: (gastric cancer OR stomach cancer OR gastric neoplasms OR gastric carcinoma OR gastric adenocarcinoma OR gastric tumor*) AND (artificial intelligence OR AI OR machine learning OR deep learning OR neural network* OR computer vision OR radiomics OR predictive analytics). The publication type was limited to Clinical Trial. No start date was specified, and the cutoff date was set to June 1, 2026. The initial search retrieved 312 records. After manual screening, one irrelevant article was excluded, and no duplicates were found. Finally, 311 articles were exported for supplementary analysis to improve the scientific rigor and comprehensiveness of our overall results.

**Figure 1:**
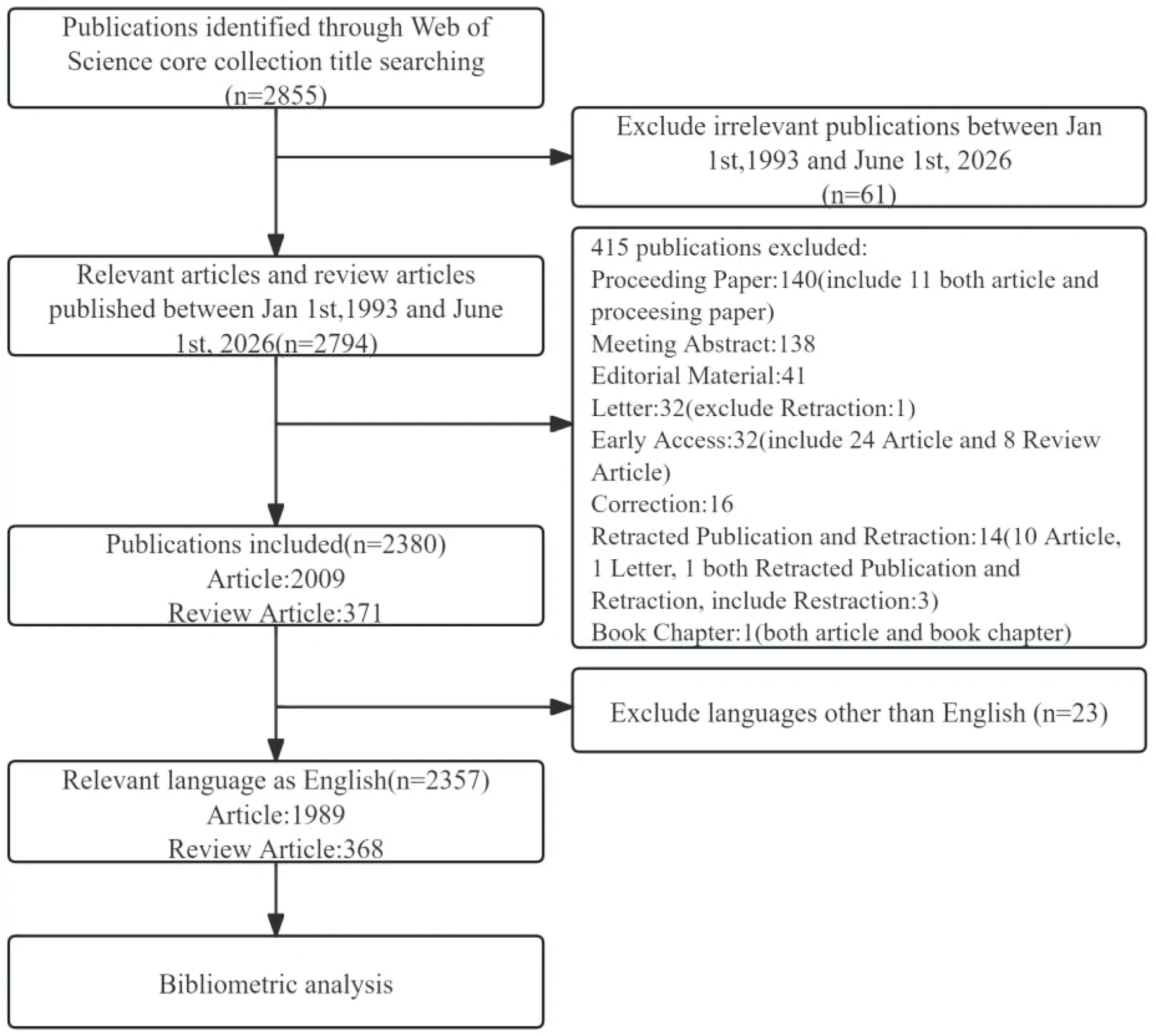
The inclusion and exclusion of publications.

### 2.2 Data processing

First, the 2,357 eligible publications were imported into VOSviewer (version 1.6.20), and no duplicate records were identified after deduplication. Separate co-occurrence network analyses were conducted for affiliated institutions, countries, authors, references, and keywords. Documents listing publication counts stratified by country were exported, and a country co-occurrence map was generated using Scimago Graphica (Beta 1.0.55) with all parameters retained as default settings. CiteSpace (7.0R) was utilized to conduct keyword clustering analysis, as well as reference and keyword burst detection. The WoSCC database was selected as the data source; the time span was set from 1993 to 2026, all analytical parameters adopted the system default values: Slice = 1, LRF = 2.5, L/N = 10, LBY = 5, and e = 1.0. Microsoft Excel 2021 was adopted to plot scatter diagrams of annual publication volumes. Subsequently, the 311 articles retrieved from PubMed were deduplicated and imported into VOSviewer and CiteSpace for comprehensive analyses. Prior to visualization, manual data cleaning was conducted. Author names, institutional affiliations, and keywords were reviewed and standardized to consolidate synonyms and remove duplicates. This process improved data quality, strengthened the accuracy and transparency of the results, and ensured the reproducibility of our findings.

## 3 Results

### 3.1 Statistical analysis of annual publication output

Analysis of retrieved records showed that the annual publication count displayed an increasing trend from 1993 to 2026. **Figure 2** presents the publication volume in this field during the last 20 years. Prior to 2015, annual publications in this field were limited, and research advanced slowly, marking the initial exploratory phase. Publication outputs rose year by year after 2016, which can be fitted by the quadratic regression equation: y=1.0055x^2^ − 22.542x + 86.144, with a coefficient of determination R^2^ = 0.7674 (> 0.7). A sharp increase occurred after 2019, and global publication output peaked at 599 papers in 2025. The global annual publication number reached a peak of 599 in 2025. The yellow dashed polynomial fitting curve presented in the figure further validated the sustained long-term growth trend of this field (R^2^ = 0.7674), revealing promising prospects for future research advances and clinical translation of AI in GC. Although the publication data for 2026 only covered records up to June, the overall output is projected to maintain a prominent upward tendency.

**Figure 2:**
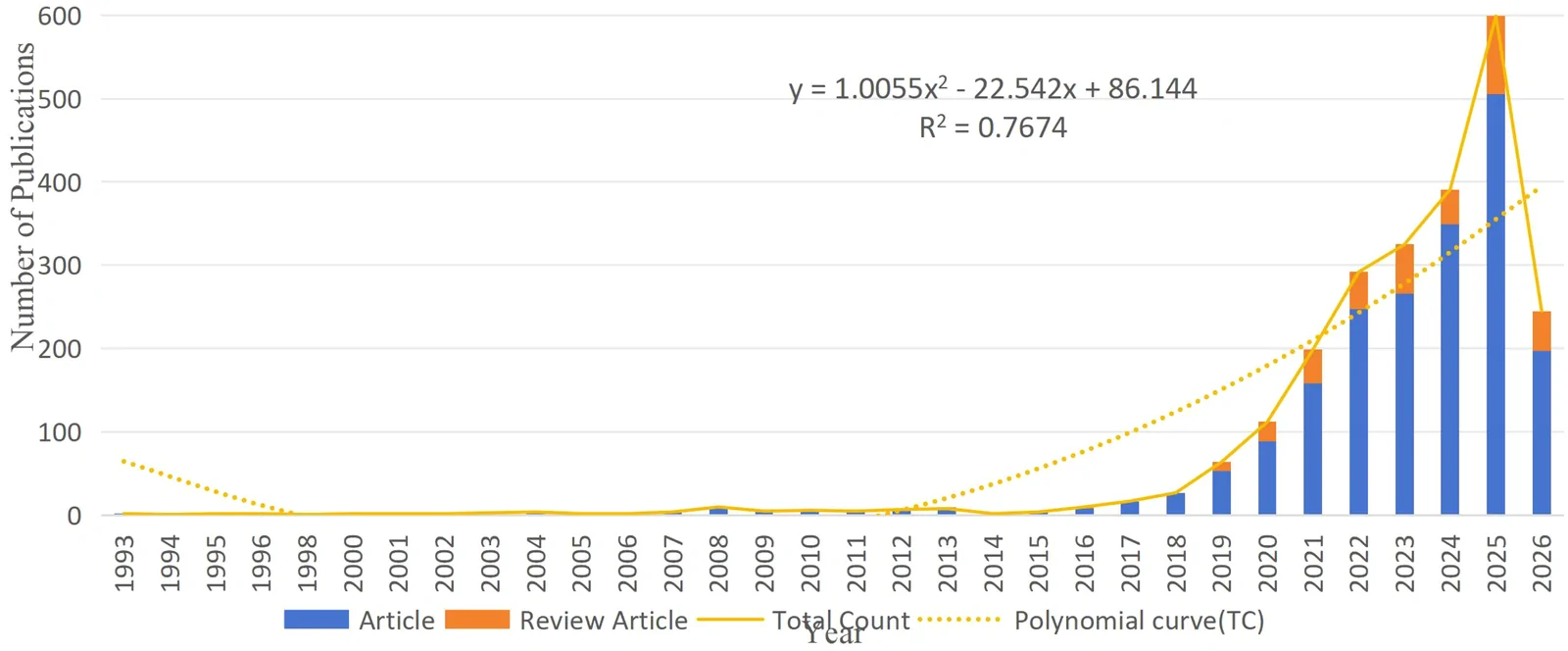
Trends of the related annual publications in WoSCC.

### 3.2 Co-authorship network analysis of authors

Co-authorship is a vital metric for evaluating research collaboration, as network maps could intuitively display researchers’ collaborative relationships and academic contributions. According to the statistical results generated by VOSviewer, a total of 13,376 authors have contributed to research on AI in GC, with an average of 5.68 authors per article. Notably, **Table 1** summarizes the publication output of the top 10 most productive authors in this field. Guoxin Li from China ranked first, with 38 publications. Other highly productive authors, including Wei Wang and Jie Tian, each contributed 30 or more publications. Among the top 10 productive researchers, nine were affiliated with Chinese institutions, and one was based in Japan. **Figure 3** presents the author collaboration network to visualize cooperative relationships among researchers. Larger nodes correspond to higher publication counts, while connecting lines stand for partnerships between authors. Larger node sizes, more connections, and darker lines within the same color group collectively reflect closer collaborative ties between authors. As shown in the figure, multiple stable core author clusters led by Li Guoxin, Wang Wei, Tian Jie and other scholars have formed in this research field. Judging from node sizes, the research teams of Li Guoxin, Wang Wei and Yu Jiang exhibit strong collaborative connections. Color coding in the network graph reveals strong collaborative connections within the research teams of Tian Jie, Fang Mengjie and Dong Di. Researchers including Huang Changming, Li Ping and Xie Jianwei have just over ten publications, yet their collaborative ties gradually deepen, with most of their publications released in the past two years. With only 25 publications, the Japanese author Tada Tomohiro has the highest citation count at 1,536, which is significantly higher than the second-highest count of 1,503. This indicates that Dr. Tada likely published impactful methodological or clinical studies.

**Table 1:** Top 10 authors.

Rank	Author	Country	Documents	Citations	Total link strength
1	Li Guoxin	China	38	1503	264
2	Wang Wei	China	31	1215	192
3	Tian Jie	China	30	1275	64
4	Dong Di	China	28	1219	62
5	Tada Tomohiro	Japan	25	1536	10
6	Yu Jiang	China	25	955	173
7	Jiang Yuming	China	24	1257	229
8	Yu Honggang	China	22	841	68
9	Wu Lianlian	China	21	790	68
10	Chen Hao	China	20	858	99

**Figure 3:**
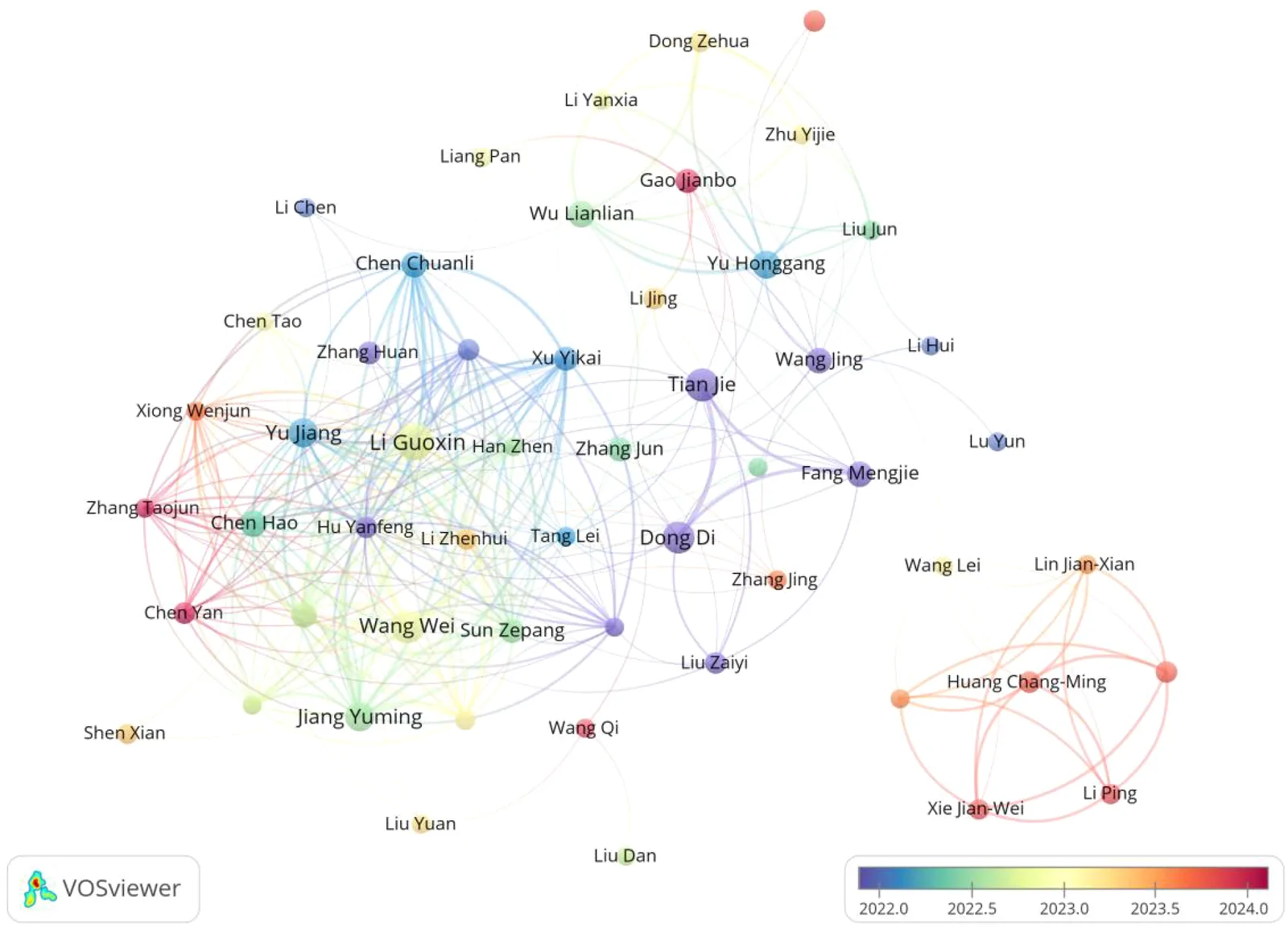
Co-authorship network collinear diagram.

### 3.3 Co-occurrence network analysis of organizations

VOSviewer analysis revealed that a total of 3115 organizations have engaged in research on AI applied to GC, 69 of which published no fewer than 15 articles each. **Table 2** summarized the publication profiles of the top 10 organizations sorted by publication count, all of which were Chinese research organizations. The Chinese Academy of Sciences produced the most publications (n=102) and recorded the highest total citation count (n=3477), reflecting its outstanding academic influence. This was followed by Sun Yat-sen University, Shanghai Jiao Tong University, Southern Medical University, and Zhejiang University. This result revealed that institutions in southern China conducted extensive relevant research on AI applied to GC, with considerable potential for future development. Institutional co-occurrence analysis visualized collaborative links between institutions. In **Figure 4**, node size corresponded to publication numbers, whereas node color intensity indicated the extent of cooperation with other institutions. **Supplementary Figure 1** lists the complete collaboration visualization map of institutions. The top 50 productive institutions included Peking University, the University of Tokyo and Seoul National University, alongside numerous medical universities represented by Southern Medical University, demonstrating the global reach of this research area. Strong cooperative ties existed within each institutional cluster. A circular heatmap of institutions was additionally generated using Scimago Graphica (**Figure 4**). Darker shades denoted higher publication outputs, and more connections represented closer and more frequent collaborations between institutions. Tight connections were found between the Chinese Academy of Sciences (CAS) and the University of the Chinese Academy of Sciences (UCAS). It should be noted that CAS is a research institute while UCAS is a university; the two are affiliated but institutionally distinct. Sun Yat-sen University and Southern Medical University likewise published abundant papers, with robust collaborative relationships across the three parties together with CAS.

**Table 2:** Top 10 productive organizations.

Rank	Organization	Documents	Proportion (%)	Citations	Total link strength
1	Chinese Academy of Sciences	102	4.33	3477	249
2	Sun Yat-sen University	92	3.90	2915	169
3	Shanghai Jiao Tong University	84	3.56	1468	86
4	Southern Medical University	79	3.35	2274	170
5	Zhejiang University	66	2.80	1037	88
6	Fujian Medical University	64	2.72	465	74
7	Zhengzhou University	63	2.67	1469	73
8	University of Chinese Academy of Sciences	61	2.59	2648	180
9	Wenzhou Medical University	59	2.50	717	70
10	Wuhan University	59	2.50	1545	58

**Figure 4:**
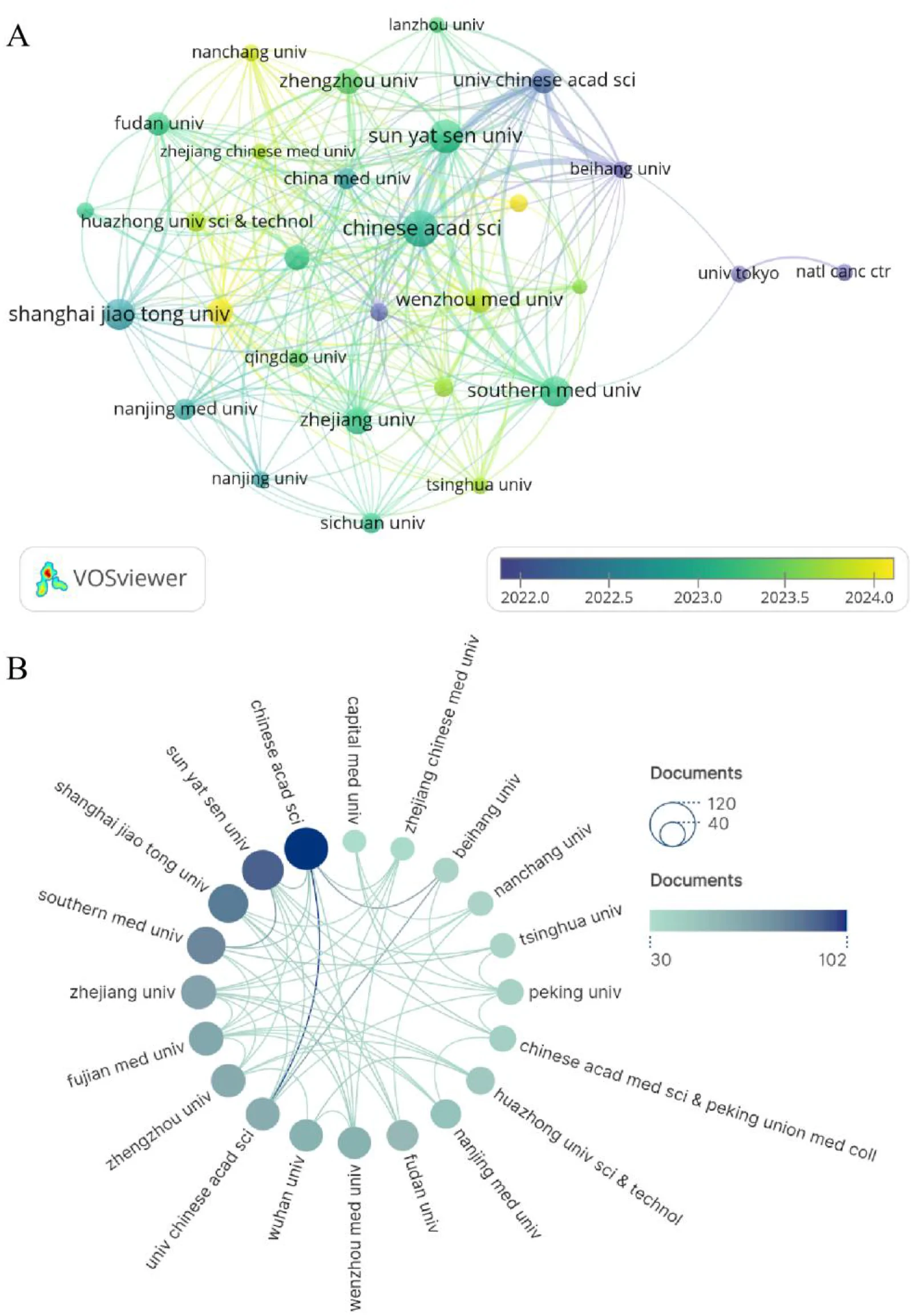
**(A)** Collaboration visualization map of institutions. **(B)** Chord diagram of organizations.

### 3.4 Co-occurrence network analysis of countries

Statistical results from VOSviewer showed that relevant publications originated from 90 countries worldwide, 44 of which released at least five articles. **Table 3** summarized metrics for the top 10 productive nations, demonstrating that the top three recorded far higher total citation counts than other global contributors. China stands as the primary contributor, accounting for 61.60% of all global publications, which constitutes more than half of the worldwide total output. **Figure 5** was constructed via VOSviewer to map the international collaboration network, illustrating China’s extensive cooperative ties with key partners including Japan, the United States and South Korea. **Supplementary Figure 2** lists the complete collaboration visualization map of country/region. A geographic collaboration network diagram was further generated using Scimago Graphica (**Figure 5**). Countries with close cooperative ties were assigned to distinct color-coded clusters according to bilateral collaboration intensity. The thickness of connecting lines represents the degree of collaboration closeness. China produced the largest number of publications and served as the primary contributor to research in this field. Although the node size of the United States was not the largest, it maintained prominent collaborative links with China. Additionally, the US established partnerships with Canada, Australia and numerous European countries, reflecting its strong capacity for international collaboration and vital bridging role across global research networks.

**Table 3:** Top 10 productive countries.

Rank	Country	Documents	Proportion (%)	Citations	Total link strength
1	People’s Republic of China	1452	61.60	22682	293
2	USA	257	10.90	6798	345
3	Japan	192	8.15	5915	129
4	South Korea	179	7.59	3195	120
5	Italy	81	3.44	1995	178
6	Germany	74	3.14	3565	197
7	England	73	3.10	3325	217
8	India	71	3.01	1136	108
9	Iran	52	2.21	599	57
10	Australia	48	2.04	975	115

**Figure 5:**
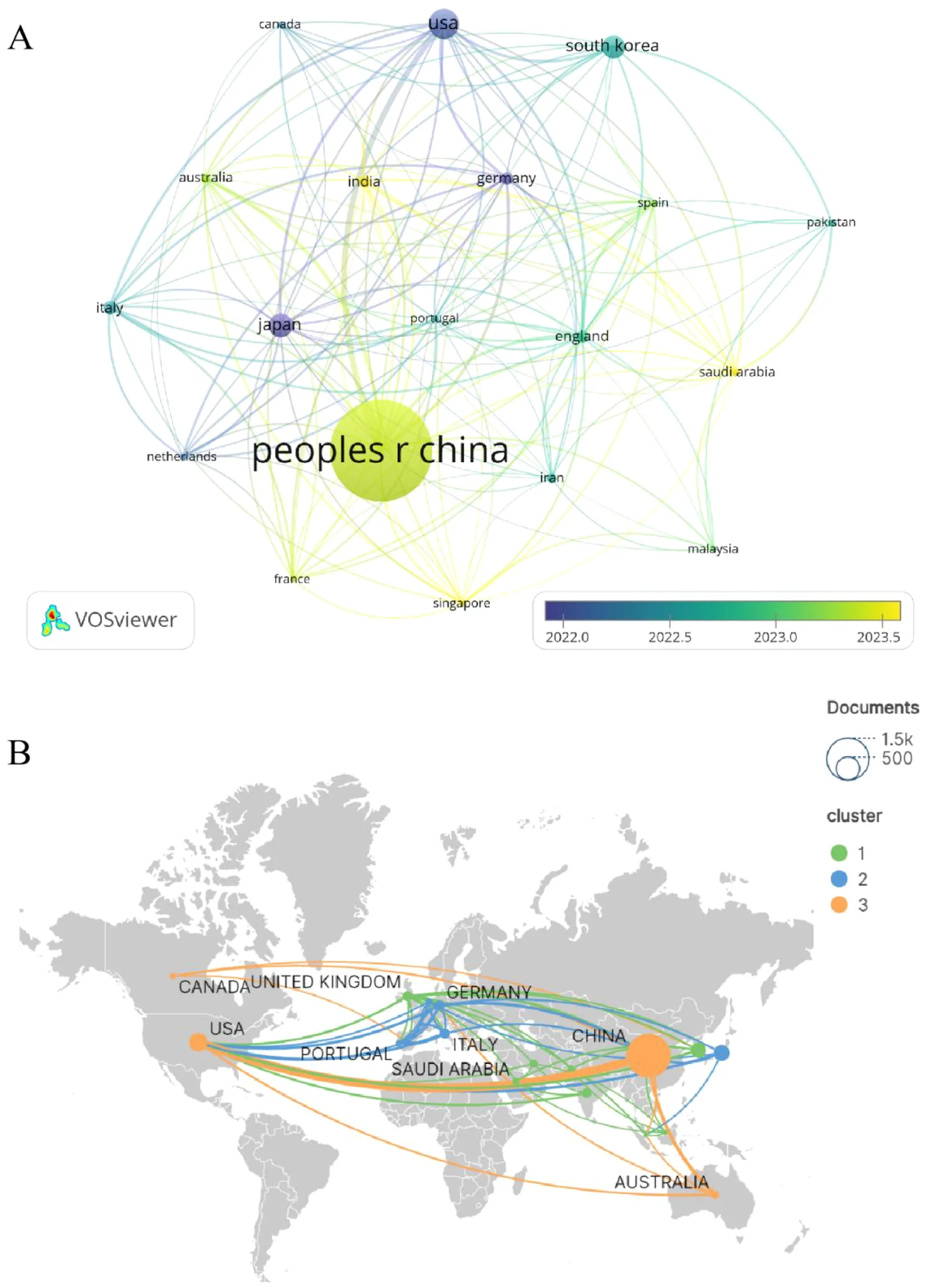
**(A)** Country/region collaboration map generated by VOSviewer. **(B)** A geographic collaboration network diagram generated by Scimago Graphica.

### 3.5 Keyword analysis

#### 3.5.1 Keyword co-occurrence network analysis

High-frequency keywords served as critical indicators reflecting the evolving research frontiers of this research field. Closely connected keywords in the network were generally grouped into clusters, each representing an emerging research theme or direction in co-word analysis. Larger circle sizes corresponded to higher keyword occurrence frequencies, while lighter colors indicated more recent publication years. The keyword co-occurrence network (**Figure 6**) was visualized using VOSviewer. The node of “gastric cancer” was the largest, followed by the relatively large nodes of “artificial intelligence” and “machine learning”. Serving as bridges within the knowledge network, these two terms linked multiple research directions including “diagnosis” and “endoscopy”. **Table 4** listed the top 10 keywords ranked separately by frequency and centrality. The frequencies of “gastric cancer”, “machine learning”, “artificial intelligence” and “deep learning” substantially outpaced all other terms, confirming them as dominant research focuses. Keywords with higher occurrence frequencies corresponded to broader research coverage, whereas those with greater centrality exerted stronger influence throughout the research domain. The centrality values of “carcinoma”, “cancer” and “expression” all > 0.3, identifying these nodes as pivotal hubs. They may represented core research topics with remarkable academic impact within this field.

**Figure 6:**
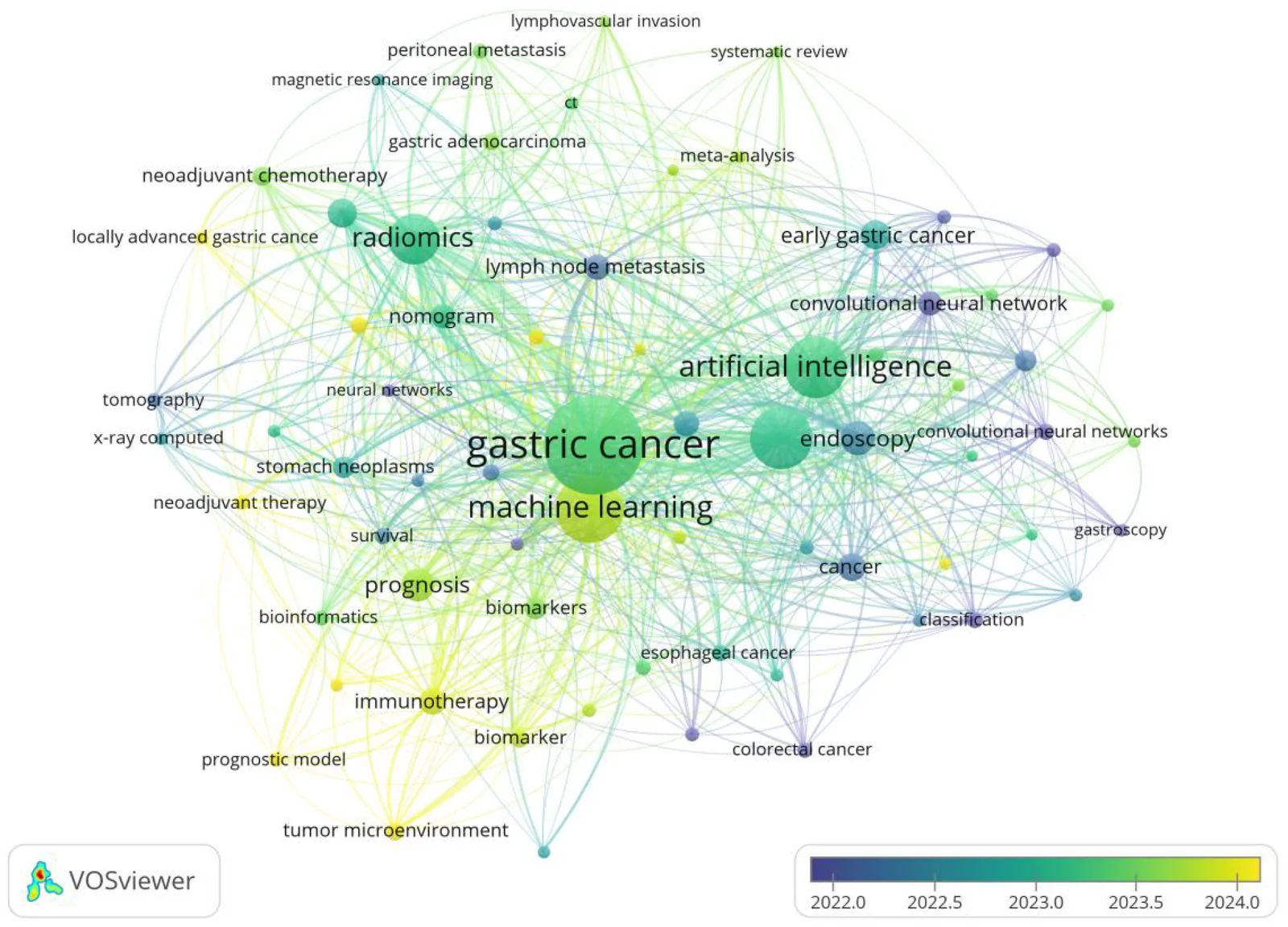
Network diagram of keywords.

**Table 4:** The top 15 keywords.

Frequency ranking					Centrality ranking				
Rank	Count	Centrality	Year	Keywords	Rank	Centrality	Count	Year	Keywords
1	1304	0.07	1996	gastric cancer	1	0.45	94	1995	carcinoma
2	449	0.02	2004	machine learning	2	0.35	159	2000	cancer
3	448	0	2018	artificial intelligence	3	0.3	168	2000	expression
4	367	0	2019	deep learning	4	0.23	60	2001	breast cancer
5	228	0.07	1996	diagnosis	5	0.2	17	2001	artificial neural network
6	183	0.04	2009	survival	6	0.2	6	1996	artificial neural networks
7	174	0.11	2006	classification	7	0.17	90	1995	colorectal cancer
8	168	0.3	2000	expression	8	0.11	174	2006	classification
9	159	0.35	2000	cancer	9	0.11	76	2003	adenocarcinoma
10	122	0.01	2019	convolutional neural network	10	0.11	75	2002	helicobacter pylori infection

#### 3.5.2 Keyword clustering analysis

Keyword clustering analysis was conducted to delineate the core research trends of AI applied in GC. The log-likelihood rate (LLR) algorithm within CiteSpace was used to generate cluster labels. Network structure was assessed via the modularity Q index, and the weighted average silhouette score S denoted the clarity of cluster divisions. As shown in **Figure 7**, the clustering network yielded a modularity Q of 0.7401 (> 0.3) and an average silhouette score S of 0.7992 (> 0.5). These findings confirm that the clustering structure exhibits satisfactory stability and reliability. The network was divided into six distinct clusters: #0 deep learning, #1 machine learning, #2 stomach neoplasms, #3 artificial intelligence, #4 gastric cancer, #5 gastrointestinal endoscopy, and #6 early gastric cancer. Clusters #0, #1 and #3 represented the AI technical framework and its fundamental applications in GC imaging analysis. Clusters #5 and #6 centered on AI-assisted endoscopic imaging for GC management, particularly early GC, corresponding to fast-growing research streams supported by substantial publications. Clusters #2 and #4 described the adoption of AI combined with CT, magnetic resonance imaging (MRI) and other imaging modalities for precision diagnosis and treatment of GC, forming the fastest-growing research hotspots in recent years. Overlapping areas and inter-node linkages demonstrate correlations across different clusters. Larger overlapping regions correspond to more shared keywords, whereas denser connections indicate stronger inter-module associations. Cluster #0 overlaps with Cluster #2, and Cluster #4 overlaps with both Cluster #1 and Cluster #5, indicating wide-ranging clinical applicability of AI in GC research.

**Figure 7:**
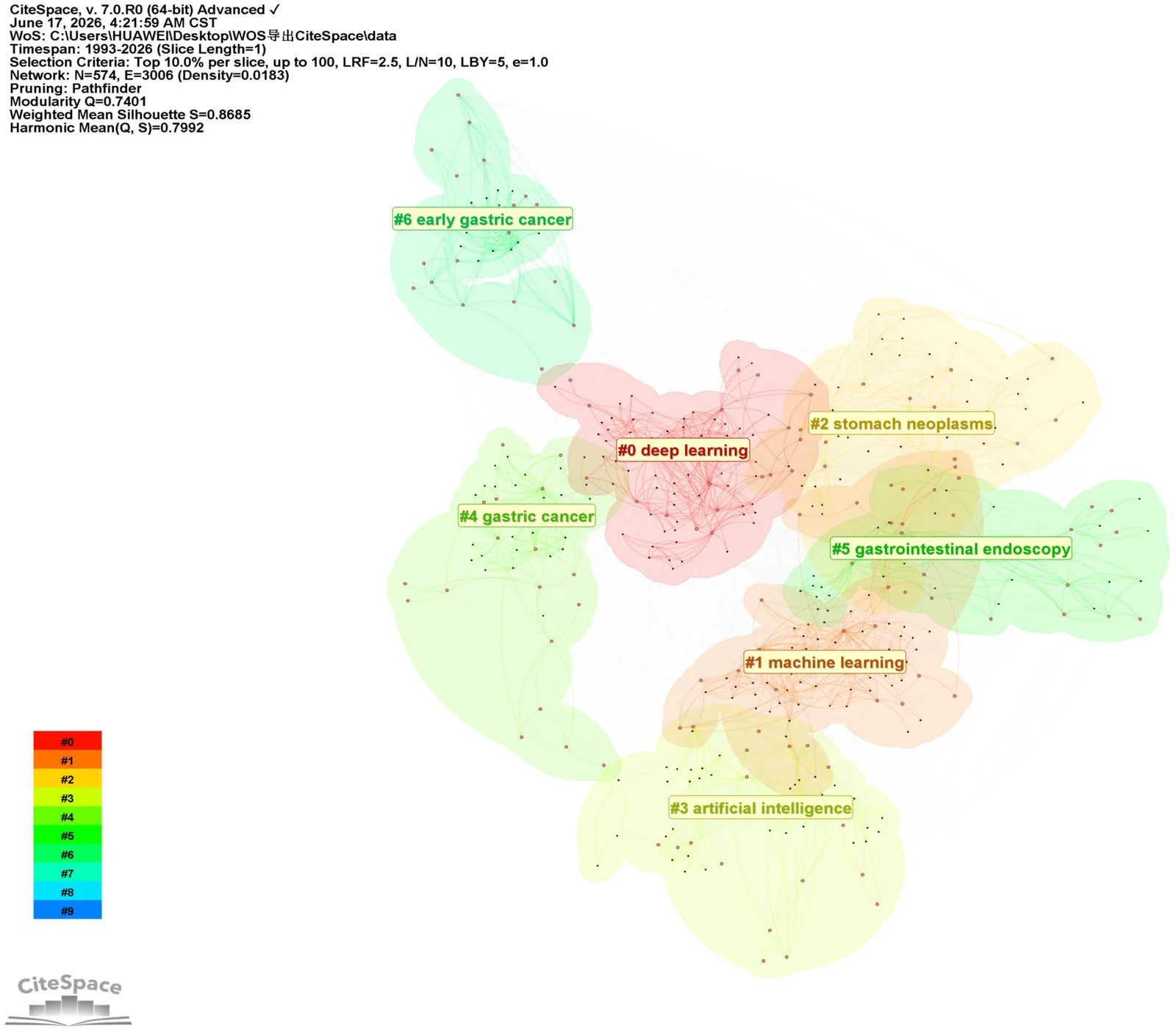
Keywords clustering diagram.

#### 3.5.3 Temporal overlay visualization of keywords clustering

The timeline view visualizes the temporal evolution of AI research in GC over the past 33 years. As shown in **Figure 8**, keywords were chronologically grouped at four-year intervals. Seven dominant clusters are displayed on the timeline, and each horizontal line corresponds to an individual keyword cluster. Each node corresponded to a single keyword, and larger node sizes indicated higher occurrence frequencies of the respective terms. The horizontal position of each node indicates the year of its first appearance in publications. Connecting lines between two nodes reflected their thematic associations; keywords with more links possessed stronger interlinkages and represented more dominant research hotspots. The largest cluster corresponds to “deep learning”. Together with the second-largest cluster “machine learning”, both fall under the methodological scope of the fourth-largest cluster “artificial intelligence”, underscoring the indispensable role of AI in GC research.

**Figure 8:**
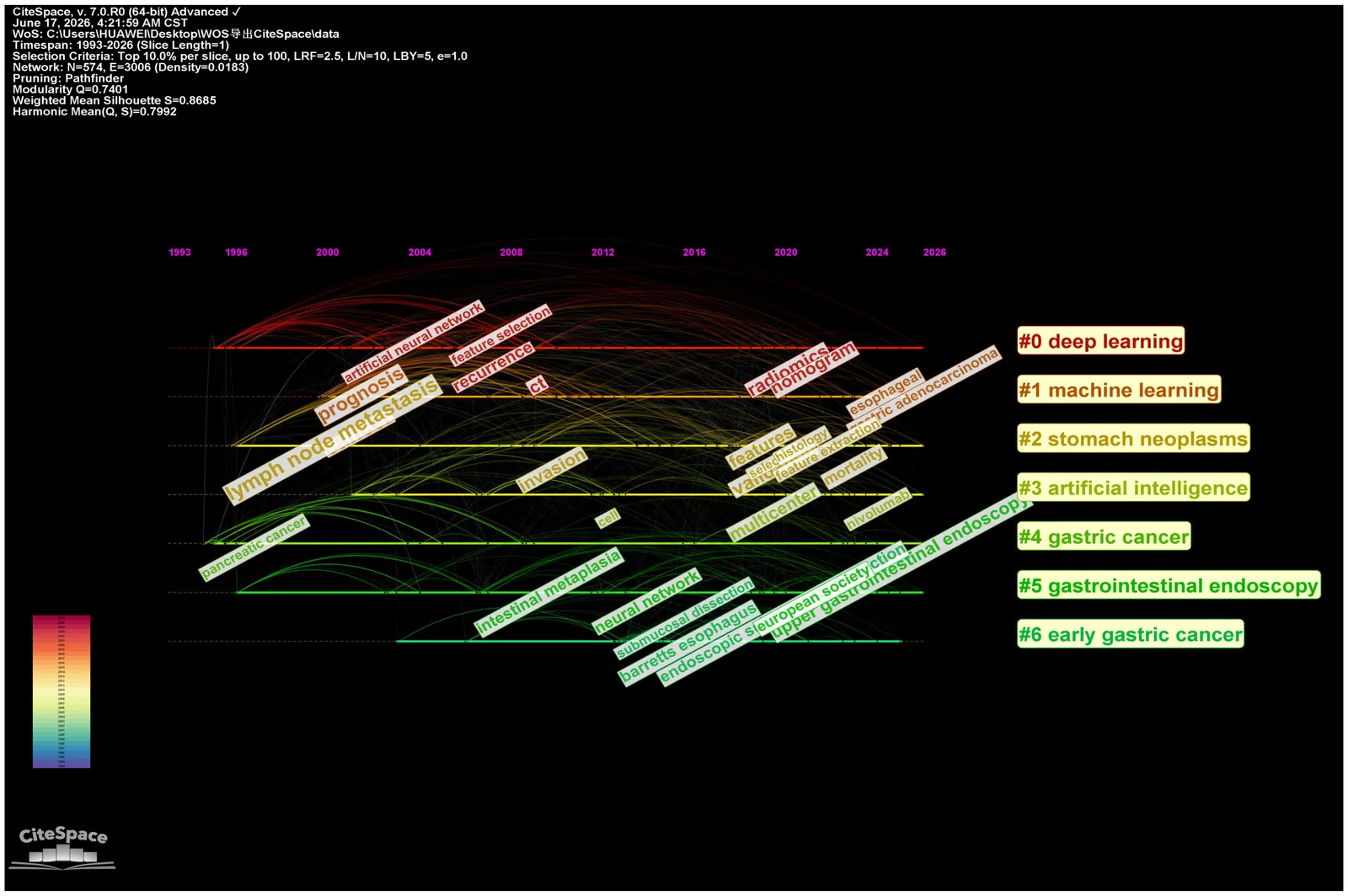
Keywords clustering time view.

#### 3.5.4 Burst detection of keyword

A citation burst represented a sudden and remarkable surge in citations obtained by one single study within a relatively short timeframe. Such phenomena generally stemmed from multiple factors that attracted widespread attention to corresponding findings, reflected by a sharp increase in citation counts. Citation bursts acted as a critical metric for assessing the academic influence of publications (**Figure 9**). Red lines denote keywords that frequently appeared during the period, while blue lines indicate relatively occasional keyword occurrences. Among all burst keywords, the term “neural networks” showed the longest burst period, ranging from 1998 to 2020. “Machine learning” sustained high burst intensity in recent years; its burst initiated in 2024 and lasted until the retrieval cutoff year of 2026, with its popularity predicted to continue growing. “Helicobacter pylori infection” possessed the highest burst strength (strength = 15.22), revealing its prominent research value and academic influence for over three years. Notably, multiple keywords such as “machine learning”, “cells”, and “recurrence” retained burst status up to 2026, signifying potential directions for subsequent research in this domain.

**Figure 9:**
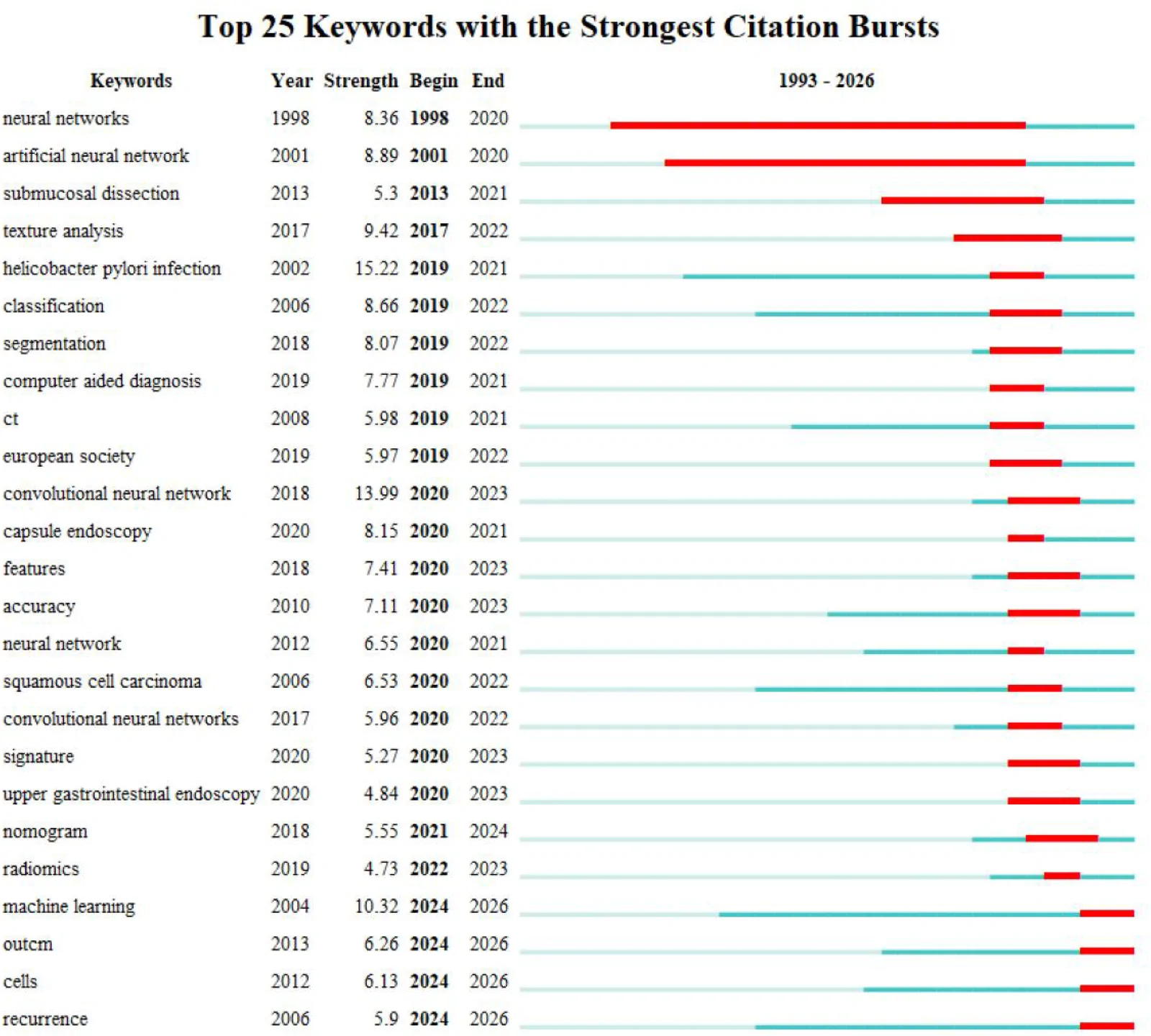
Top 25 keywords with the strongest citation bursts.

### 3.6 Co-cited reference analysis

Co-cited references represented pairs of publications cited simultaneously by other papers. Between 1993 and 2026, this study identified a total of 75,856 co-citation links from research on AI in GC, and 86 individual references earned over 50 co-citation links apiece. **Table 5** listed the top 10 references ranked by co-citation strength. Among the highly cited publications, “Global Cancer Statistics 2020: GLOBOCAN Estimates of Incidence and Mortality Worldwide for 36 Cancers in 185 Countries” (8) ranks first with a citation count of 549, followed by “Global cancer statistics 2018: GLOBOCAN estimates of incidence and mortality worldwide for 36 cancers in 185 countries” (9), which has 255 citations. Such high citation counts demonstrate the strong academic value and widespread recognition of relevant studies. Notably, the two most highly cited articles were both published in *CA: A Cancer Journal for Clinicians*. The article entitled “Gastric cancer” (10) published in The Lancet ranks third, with 249 citations. For references with no fewer than 50 co-citations, a co-citation reference network (**Figure 10**) and a density map (**Figure 10**) were constructed. In the co-citation network, node size represents co-citation frequency, and connecting lines indicate co-citation relationships between publications. The thickness and density of links reflect the strength of academic associations. In the density plot (**Figure 10**), darker coloring and larger nodes correspond to higher co-citation frequency and greater scholarly impact. Classic studies by sung h (2021, ca-cancer j clin), smyth ec (2020, lancet), and hirasawa t (2018, gastric cancer) show strong co-citation associations, underscoring their prominent academic status in AI-based GC research, as visualized in **Figure 10**.

**Table 5:** Top 10 cited references.

Rank	Author	Year	Journal	DOI	Citations	Total link strength
1	Sung H	2021	CA-CANCER J CLIN	10.3322/caac.21660	549	1726
2	Bray F	2018	CA-CANCER J CLIN	10.3322/caac.21492	255	964
3	Smyth EC	2020	LANCET	10.1016/s0140-6736(20)31288-5	249	750
4	Hirasawa T	2018	GASTRIC CANCER	10.1007/s10120-018-0793-2	244	1503
5	Bray F	2024	CA-CANCER J CLIN	10.3322/caac.21834	160	339
6	Zhu Y	2019	GASTRONINTEST ENDOSC	10.1016/j.gie.2018.11.011	148	1205
7	He KM	2016	PROC CVPR IEEE	10.1109/cvpr.2016.90	143	568
8	Lambin P	2017	NAT REV CLIN ONCOL	10.1038/nrclinonc.2017.141	139	684
9	Bass AJ	2014	NATURE	10.1038/nature13480	137	471
10	Gillies RJ	2016	RADIOLOGY	10.1148/radiol.2015151169	132	663

**Figure 10:**
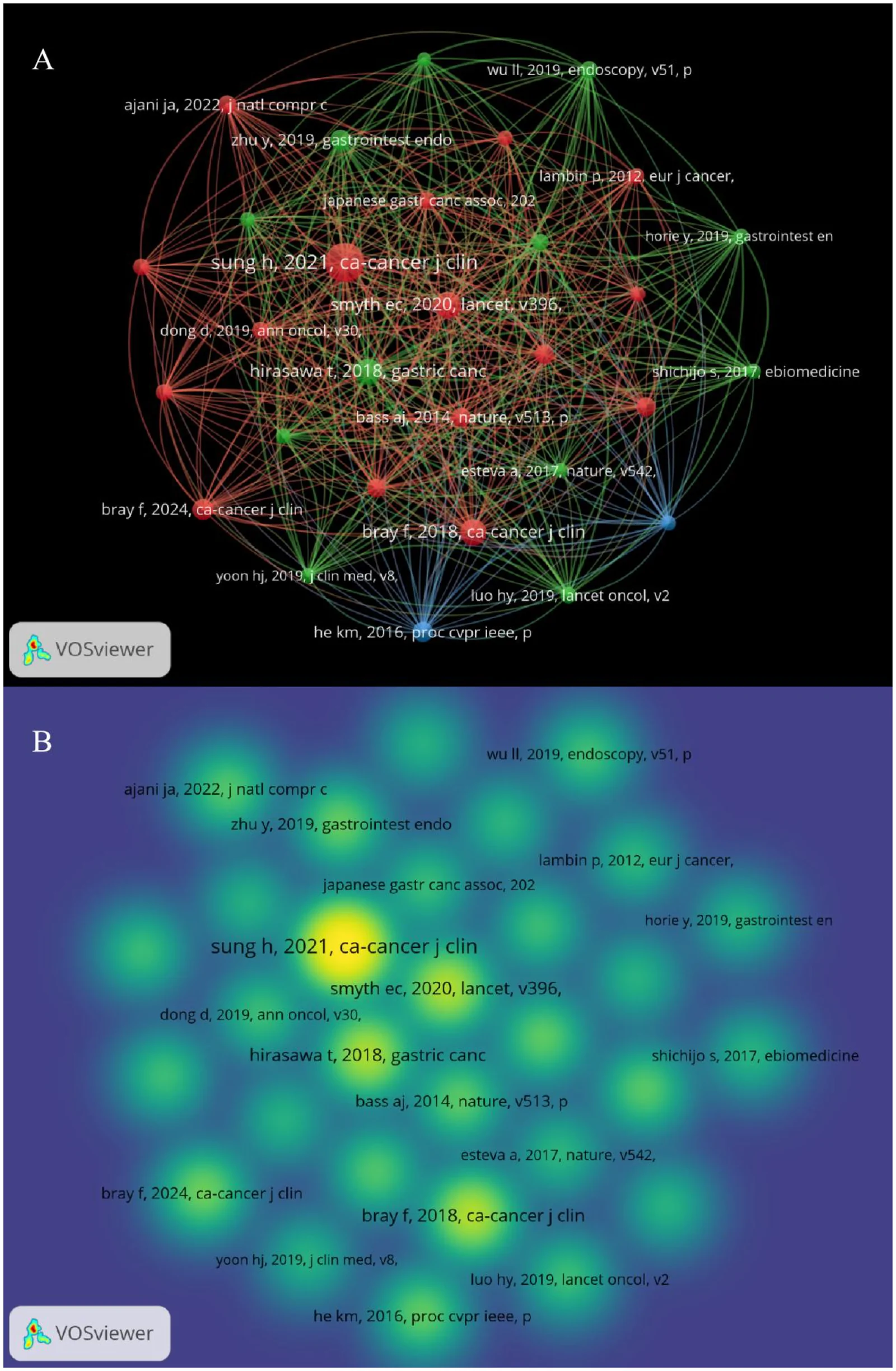
**(A)** Co-cited references network; **(B)** A density map.

### 3.7 Burst detection of co-cited references

A citation burst represented a sharp surge in citation counts of publications within a specific period, which partly reflected the developmental dynamics of a research field. In this study, **Figure 11** displayed the top 25 references with the strongest citation bursts identified via CiteSpace. Citation bursts of these references emerged as early as 2016, with burst strengths ranging from 16.67 to 99.79. The article titled Global Cancer Statistics 2020: GLOBOCAN Estimates of Incidence and Mortality for 36 Cancers Across 185 Countries by Song et al., published in CA: A Cancer Journal for Clinicians (8), recorded the highest burst strength of 99.79, and its burst duration extended from 2021 to the retrieval year 2026. The work “Gastric Cancer, Version 2.2022” authored by Ajani JA et al. (11) presented a burst strength of 35.57, representing the highest burst strength among references with active bursts in the latest five years. Its burst started in 2024 and lasted until 2026. Despite its current strength of 35.57, this reference was projected to attract more citations from 2026 onward, indicating a favorable citation trend.

**Figure 11:**
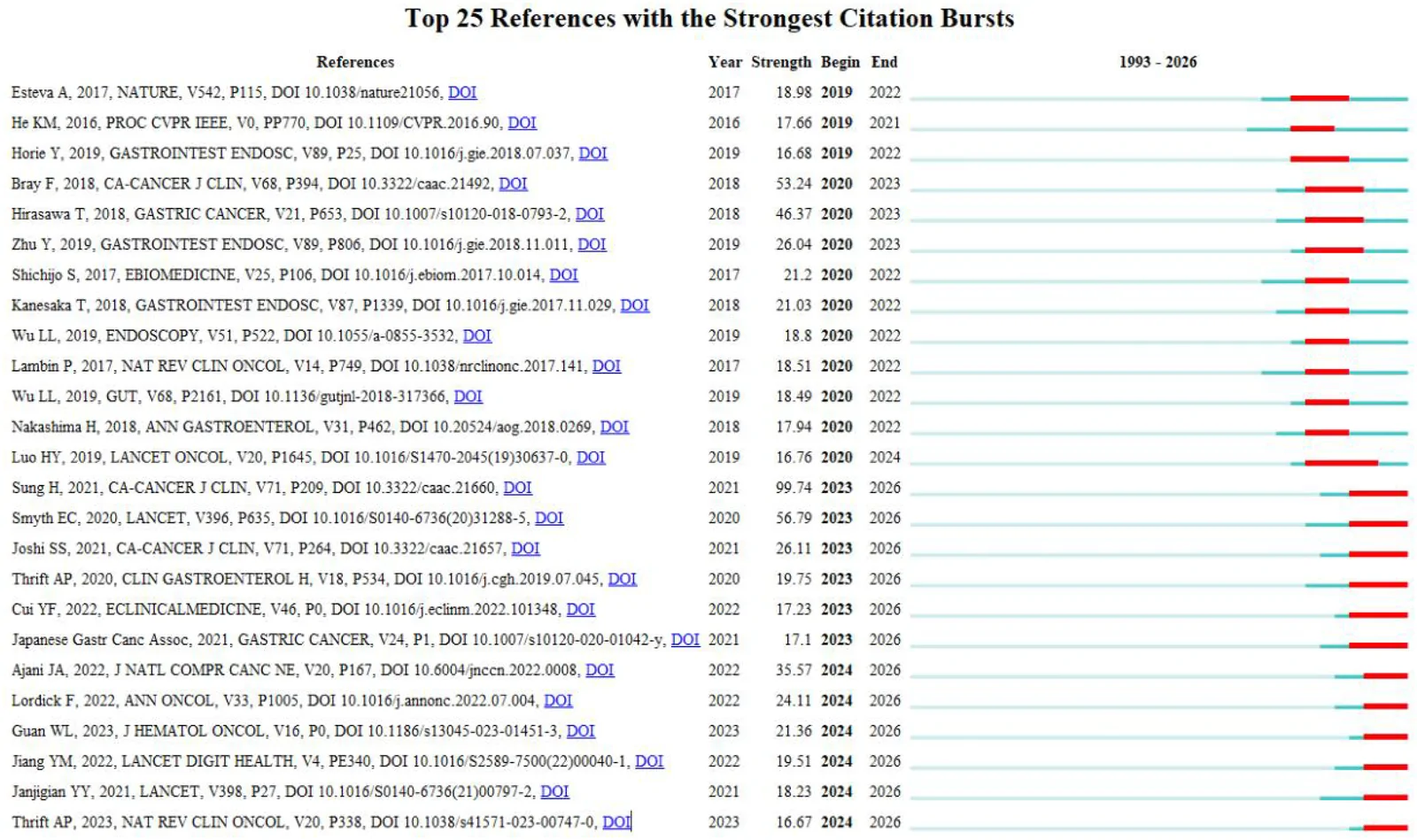
Top 25 references with the strongest citation bursts.

### 3.8 PubMed clinical trial supplementary analysis

A statistical chart of the total number of publications of 311 Clinical Trial articles in the PubMed database was generated using Microsoft Excel (**Figure 12**). The results showed that the total publication volume exhibited an exponential growth trend, with the fitting equation of (y = 0.8331e^0.0974x^) and the coefficient of determination (R^2^ = 0.8663), indicating a good goodness of fit. Substantial growth was detected across 2017–2019 and 2023–2025, with two prominent peaks over the past decade in 2019 (n = 25) and 2025 (n = 35) respectively. Although the data for 2026 are still incomplete (covering only the first 6 months prior to retrieval), the existing trend suggests that the research output in this field may continue to maintain a growth trend. When compared with the publication volume statistical chart of the WoSCC, both charts consistently show that in the early period (around the year 2000), the number of relevant studies was small and grew relatively slowly; in contrast, the number of studies has increased significantly over the past decade.

**Figure 12:**
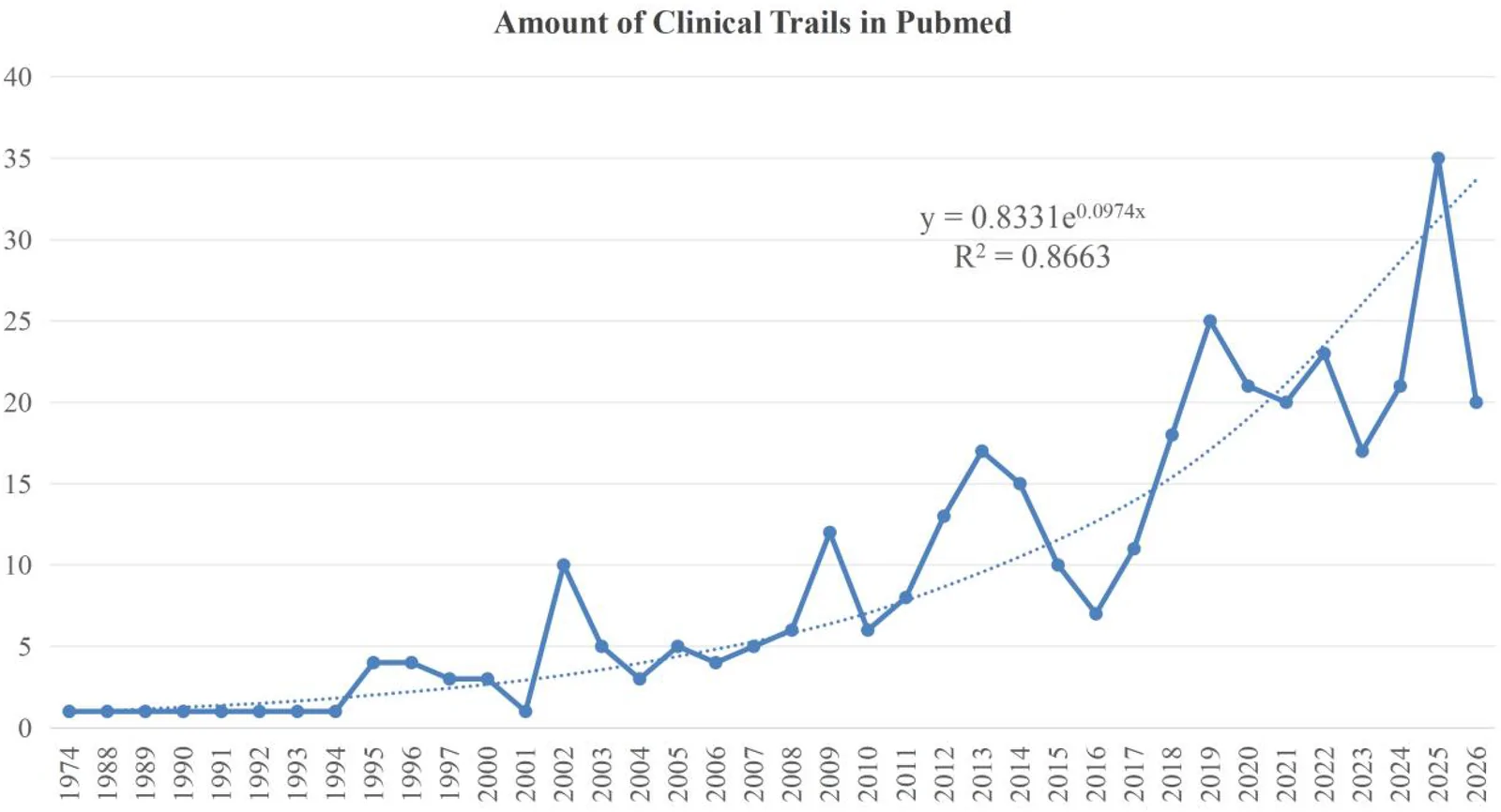
Trends of the related annual publications in Pubmed.

Two graphs were generated for keyword analysis: a keyword co-occurrence network (**Figure 13**) via VOSviewer and a burst plot of the top 10 keywords (**Figure 14**) using CiteSpace. By comparing the keyword co-occurrence maps of the PubMed database (**Figure 13**, **Table 6**) and the WoSCC database (**Figure 6**, **Table 4**), similarities and discrepancies between the two datasets were identified. Comparison between **Figures 6**, **13** revealed that the node labeled gastric cancer was the largest with abundant connecting lines in both visualizations, indicating that GC serves as the core research focus of this field. Lighter node colors correspond to more recent publication years. Keywords including “radiomics”, “immunotherapy”, and “tomography” were observed in both maps. Additionally, “chemotherapy” and “phase 1” trials stood out prominently in **Figure 6**, highlighting the clinical attributes of WoSCC-included studies, which are likely to emerge as key research priorities in the future. Among the top 10 keywords, “gastric cancer” and “artificial intelligence” were shared by the WoSCC dataset. The terms “advanced gastric cancer” and “chemotherapy” were distinctive keywords within clinical trial publications. Notably, “her2” represented a biomarker, molecular research is expected to become a prospective research direction.

**Figure 13:**
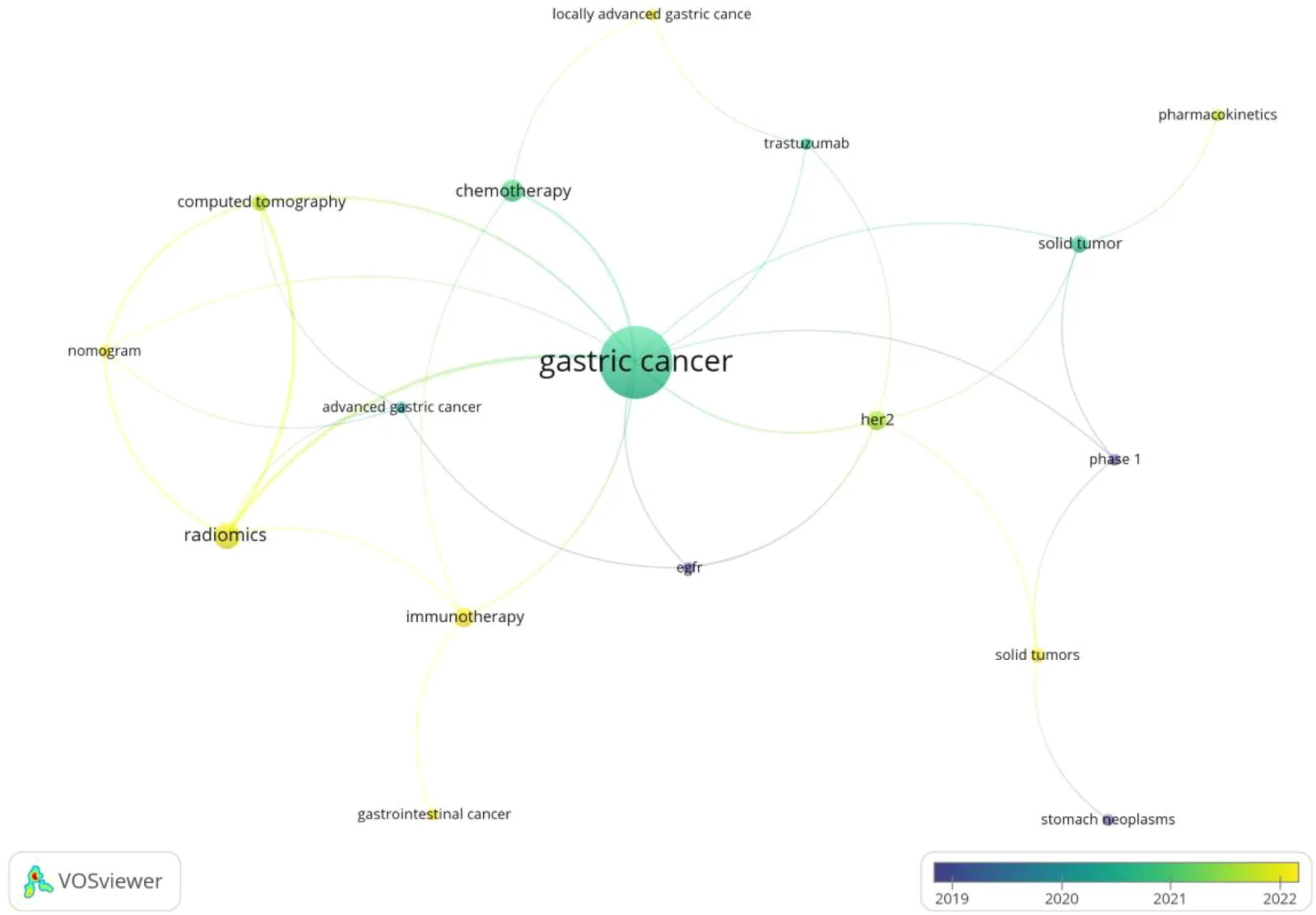
Network diagram of keywords in Pubmed.

**Figure 14:**
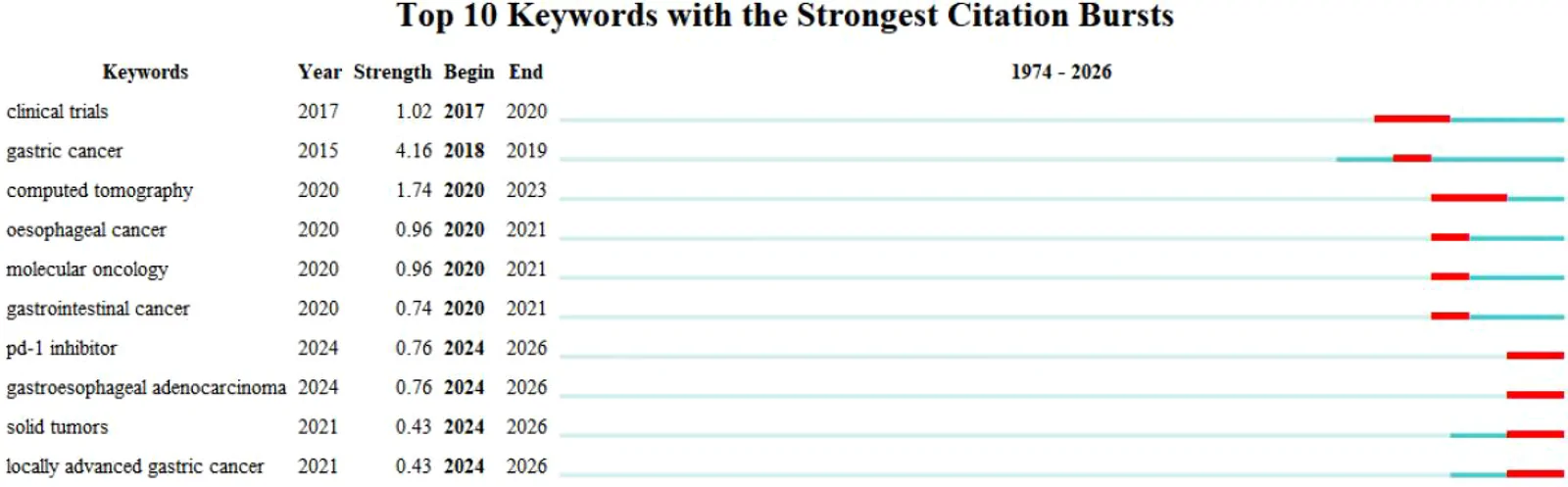
Top 10 keywords with the strongest citation bursts.

**Table 6:** Top 10 keywords in Pubmed.

Rank	Keyword	Occurrences	Total link strength
1	gastric cancer	36	22
2	radiomics	9	16
3	chemotherapy	7	5
4	artificial intelligence	6	0
5	her2	6	7
6	immunotherapy	6	5
7	computed tomography	5	11
8	solid tumor	5	4
9	solid tumors	4	3
10	advanced gastric cancer	3	4

Burst detection maps of keywords were generated via CiteSpace software (**Figure 14**). Comparative analysis with the keyword burst map of the WoSCC database (**Figure 9**) revealed that the overall burst strength of keywords in **Figure 14** was lower than that in **Figure 9**, which indicated that research within this field remains in the developmental stage. The term “molecular oncology” in **Figure 14** addressed overlapping research themes with “texture analysis” in **Figure 9**. Likewise, “submucosal dissection” in **Figure 9** shared a comparable research focus with “upper gastrointestinal endoscopy” in **Figure 14**. **Figure 14** reveals that research hotspots have gradually shifted toward clinical applications in recent years. Among the burst keywords identified, clinical trials (2017–2020) and gastric cancer (2018–2019) represented the early emerging terms. Gastric cancer exhibited the maximum burst strength (Strength = 4.16), reflecting extensive research attention focused on GC during this period. Subsequently, computed tomography served as a long-lasting burst keyword spanning 2020 to 2023, underscoring the vital role of medical imaging technology in AI-assisted research on GC. Furthermore, despite the relatively low burst strengths of keywords such as pd-1 inhibitor (Strength = 0.76) and solid tumors (Strength = 0.43), their latest burst period (2024–2026) implies that these topics may suggesting considerable opportunities for further investigation.

## 4 Discussion

Bibliometric analysis is a quantitative approach for characterizing the knowledge structure, identifying research hotspots, and elucidating the developmental trajectories of a specific research field. In this study, VOSviewer and CiteSpace were used to comprehensively characterize the research landscape and evolution of AI applications in GC over the past 33 years. Keyword co-occurrence, clustering, and burst detection analyses were performed to identify major research directions, characterize the evolution of research hotspots, and explore potential future trends. To improve the comprehensiveness and robustness of the analysis, data from the WoSCC and PubMed databases were incorporated for complementary analyses. Over the 33-year study period, the annual number of publications on AI applications in GC showed an overall increasing trend, reflecting sustained and growing research interest in this field. Based on the integrated findings of the present analysis, a schematic timeline (**Figure 15**) was constructed to illustrate the evolution of major research themes. Notably, 2017 and 2023 emerged as two important turning points, corresponding to distinct shifts in research focus. The timeline summarizes the predominant research hotspots across different periods and provides a cautious perspective on the potential future development of AI applications in GC.

**Figure 15:**
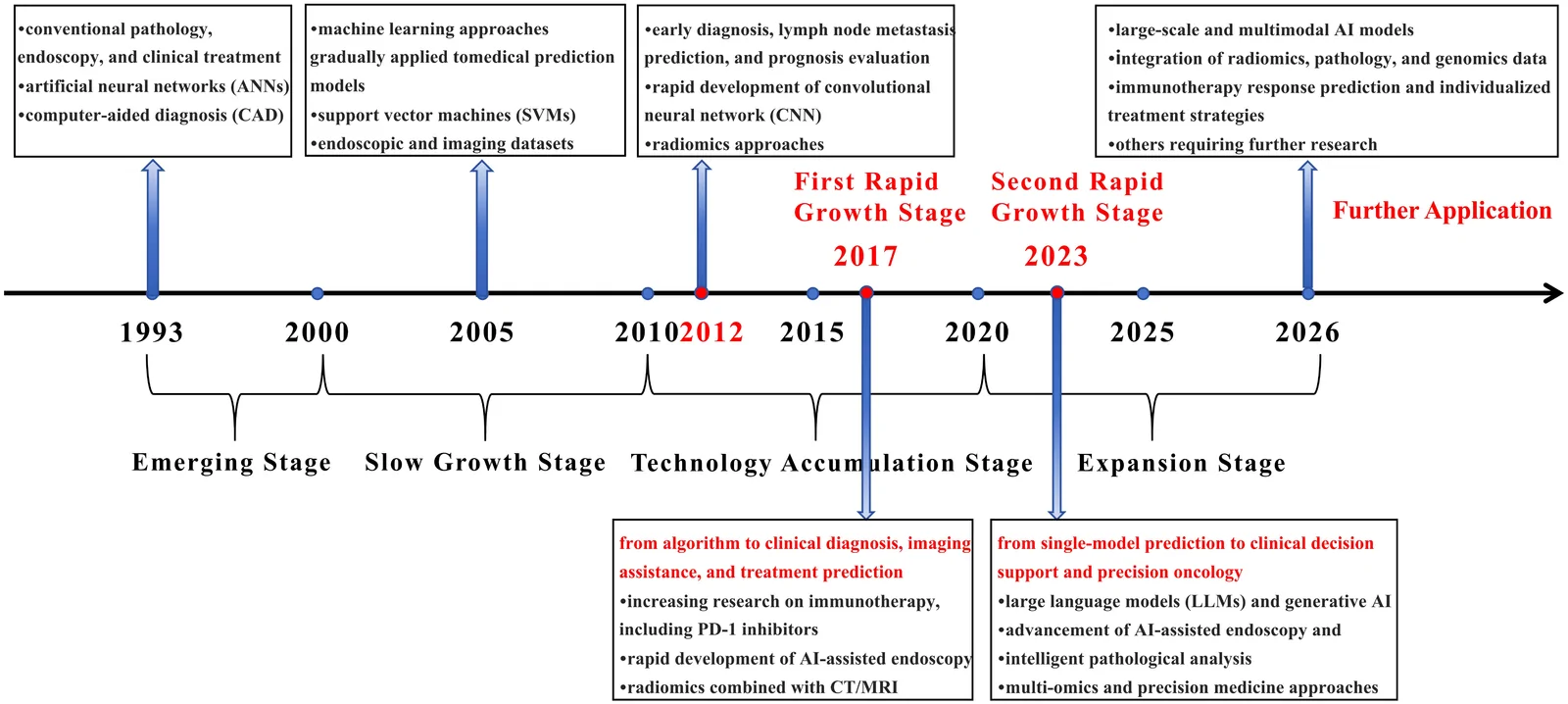
Schematic diagram of hotspots and trends.

### 4.1 Collaboration network analysis

Author collaboration network analysis showed that nine of the top ten productive authors were from China. A stable academic collaboration network has been formed among core researchers, including Guoxin Li, Wei Wang, and Jie Tian, reflecting the rapid development and robust research capacity of China in this field. Although Japanese scholar Dr. Tomohiro Tada published only 25 articles, he achieved the highest total citation count (1,536 citations), which exceeded the 1,503 citations of other leading active authors. This finding indicates that Dr. Tada’s work contains highly influential methodological and clinical studies that have shaped the development of the research field. Overall, the research landscape of AI in GC may has gradually established a mature collaborative pattern centered on prolific authors and core research teams.

Institutional collaboration network analysis further revealed that all of the top ten most productive institutions focused on AI applications in GC are located in China. This dominant domestic contribution can be attributed to the high disease burden of GC nationwide, abundant clinical data resources, and continuous financial support for interdisciplinary research integrating AI and clinical medicine. Leveraging multidisciplinary platform advantages, representative institutions including the Chinese Academy of Sciences, Sun Yat-sen University, and Southern Medical University have developed strong research competence in medical imaging, computer technology, and clinical translational applications. Furthermore, intensive cooperation among core institutions demonstrates that this field may be gradually transitioning from single-center investigations to multi-institutional collaborative research, which substantially improves the translational potential and clinical applicability of AI-related GC research.

National collaboration network analysis demonstrated that China is the predominant contributor to global AI research on GC, accounting for 61.60% of total publications. China maintains robust collaborative ties with Japan, the United States, and South Korea, reflecting highly consistent clinical needs and research priorities across gastric cancer-endemic areas in Asia. Notably, despite its lower publication output relative to China, the United States functions as a critical bridging node in the international collaboration network. This unique collaborative strength can be attributed to its advanced AI infrastructure, mature computer science systems, and extensive global research partnerships.

### 4.2 Keyword analysis

Keyword co-occurrence network analysis revealed that the research hotspots of AI in GC are primarily centered on themes including “gastric cancer”, “artificial intelligence”, “machine learning”, and “deep learning”. This research trend can be attributed to the rapid accumulation of medical imaging, endoscopic, and pathological data in recent years, which has underpinned the development of intelligent diagnosis and predictive models. Meanwhile, keywords such as “diagnosis” and “endoscopy” exhibit strong correlativity, indicating that AI-assisted early screening and diagnosis represent the current research priority, which closely aligns with clinical diagnostic and therapeutic demands. Furthermore, high-centrality keywords including “carcinoma” and “expression” suggest that the research focus may be gradually expanding toward tumor feature recognition and precision diagnosis and treatment.

Keyword cluster analysis further indicated that AI research in GC predominantly focuses on deep learning, machine learning, and intelligent endoscopy. Such advancements are largely driven by the progress of radiomics and multimodal data analysis, which enable AI models to extract latent tumor features that are undetectable via conventional imaging methods.

Keyword temporal evolution analysis further illustrated the dynamic developmental trends of AI research in the field of GC. Early studies predominantly focused on the construction and optimization of AI algorithms and models. Furthermore, the high clinical demand for early GC diagnosis has driven the rapid application of AI techniques to improve diagnostic efficiency and accuracy. Traditional diagnostic strategies rely heavily on physician experience, which inevitably introduces subjective bias, thereby highlighting the necessity and superiority of AI-assisted diagnostic tools in clinical practice.

Burst detection of keyword analysis revealed that the research hotspots of AI in GC have evolved from fundamental algorithm research toward clinical application expansion. The prominent early burst of “neural networks” indicated that artificial neural networks (ANNs) constituted the core technical foundation for early AI exploration in this field. The sustained burst of “machine learning” in recent years suggests that data-driven analytical methods have become a dominant research direction, which can be explained by the rapid accumulation of medical imaging data, electronic medical records, and multi-omics data. In addition, “Helicobacter pylori infection” exhibited the highest burst intensity, identifying it as a critical risk factor for GC initiation and progression. Notably, the ongoing burst of “recurrence” in recent years demonstrates increasing and continuous scholarly attention to GC prognosis.

### 4.3 Reference analysis

Co-citation analysis revealed that AI research pertaining to GC has gradually evolved into an integrated framework that combines disease research with intelligent diagnosis and treatment. Among cited publications, the GLOBOCAN cancer statistics and GC investigations published in *The Lancet* yielded the highest co-citation frequencies. This finding demonstrates that the substantial disease burden and unmet clinical demands of GC act as core drivers propelling the progression of AI research in this field. Meanwhile, the landmark work by Hirasawa et al. concerning AI-assisted endoscopic diagnosis of GC has received extensive citations, indicating that the utility of AI in early GC identification and auxiliary management has gained growing recognition and clinical validation.

Burst detection of co-cited references further indicated that the GLOBOCAN global cancer statistics presented the strongest citation burst strength, demonstrating that global epidemiological trends constitute a fundamental basis for advancing gastric cancer-related AI research. In recent years, the clinical guideline *Gastric Cancer, Version 2.2022* published by Ajani et al. has received sustained research attention, reflecting that standardized diagnostic and therapeutic guidelines exert a profound impact on GC research and clinical practice.

### 4.4 Supplementary analysis

Supplementary analysis of PubMed clinical trials indicated that AI research in GC was gradually transitioning from fundamental algorithm development to clinical translational application. Although PubMed contained fewer clinical trial publications compared with the WoSCC database, it exhibited a remarkable growing trend in recent years, which reflected the rapid translational progression of AI technology from basic exploration to clinical implementation in the field of GC. Studies retrieved from the WoSCC database predominantly focused on AI technology development, imaging analysis, and predictive model construction, highlighting the applications of AI in early GC screening, auxiliary diagnosis, and risk prediction. In contrast, literature from PubMed Clinical Trials centered on keywords such as “cancer”, “tumor”, and “chemotherapy”, which were closely associated with clinical treatment, therapeutic efficacy evaluation, and prognostic management of GC. The complementary analysis of the two databases enabled a more comprehensive and systematic elucidation of the overall developmental landscape of AI research in GC.

Keyword analysis further demonstrated that PubMed-based studies focused on clinically relevant topics, including “chemotherapy”, “her 2”, and “advanced gastric cancer”, which reflected the increasing clinical emphasis on AI-assisted treatment decision-making, therapeutic response prediction, and precise patient stratification. As a critical molecular biomarker for GC, her 2 was strongly correlated with targeted therapy selection, making it a promising direction for AI-enabled precision diagnosis and treatment. Furthermore, the consistent appearance of “computed tomography” and “radiomics” indicated that medical imaging and multimodal data remained core foundations for the clinical translation of GC AI research. The abrupt emergence of immune-related keywords such as “pd-1 inhibitor” further revealed that the combination of AI and immunotherapy for efficacy prediction and patient screening would become a pivotal research frontier.

Clinical studies have revealed that the clinical applications of AI and radiomics in GC predominantly focus on early screening, auxiliary diagnosis, therapeutic response prediction, and clinical decision support. Computer-Aided Diagnosis (CAD) systems are adopted for gastric tumor detection and can markedly improve the detection rate of endoscopic lesions (12). Real-time computer-aided optical diagnostic techniques further boost instant diagnostic performance for gastric neoplasms (13). AI-based quality control systems help improve the quality of gastrointestinal endoscopic examinations (14). AI models constructed based on endoscopic images support remote screening for Helicobacter pylori infection and gastric precancerous lesions (15). AI-assisted endoscopic reporting systems narrow diagnostic discrepancies among endoscopists with varying experience and enhance consistency in lesion description and final diagnosis (16). CT-based radiomics signatures are applied to predict the prognosis of patients with advanced GC (17). CT, MRI and their combined radiomic models are utilized to evaluate pathological response after neoadjuvant chemotherapy (18), while CT radiomics also facilitates evaluating radiotherapy response (19). Collectively, AI and radiomics are driving GC diagnosis and treatment toward precision, digital intelligence and personalized management. These findings are consistent with the results derived from the WoSCC database analysis, which verifies the robustness of the present conclusions.

### 4.5 Application of AI in GC

#### 4.5.1 Overview of GC

GC ranks fifth globally in terms of incidence and mortality, posing a severe threat to human health. It develops progressively from precancerous lesions including chronic atrophic gastritis, intestinal metaplasia and dysplasia. Most patients are diagnosed at advanced stages owing to the absence of specific early symptoms and signs, which leads to poor overall prognosis for these patients (20). Early diagnosis creates a critical window for early intervention; timely treatment can delay or even reverse disease progression, especially among high-risk populations (21).

Major risk factors for GC encompass Helicobacter pylori infection, dietary habits, obesity, smoking and genetic susceptibility. Comprehensive diagnostic workflows facilitate early detection. Pathological confirmation of GC relies on endoscopic biopsy, while CT, endoscopic ultrasonography (EUS), positron emission tomography (PET) and laparoscopy are routinely applied for tumor staging. Endoscopic resection serves as the primary therapeutic modality for early GC. For locally advanced GC, radical surgical resection (particularly D2 lymphadenectomy) combined with adjuvant chemotherapy and radiotherapy constitutes the standard treatment strategy (22). ML, especially DL and neural networks, has demonstrated considerable potential for improving GC diagnosis, treatment and prognostic prediction. AI applications in GC has accelerated the advancement of precision diagnosis and treatment.

#### 4.5.2 Overview of AI and medical imaging

AI is a long-standing concept whose theoretical framework originated in the 1940s, and the formal term “artificial intelligence” was coined by John McCarthy in 1956. AI refers to computer algorithms capable of simulating human intelligence characteristics such as problem-solving and learning capabilities. Over the past decade, AI applications built on ML algorithms have yielded unprecedented progress in medical computer vision, covering disease diagnosis, image segmentation and prognostic outcome prediction (23). The evolution of AI from Version 1.0 to Version 4.0 indicates that this field is on the verge of profound transformation (24).

CT and MRI provide elaborate tumor information owing to their superior spatial resolution and tissue contrast (25). Nevertheless, conventional imaging modalities possess inherent shortcomings in detecting minute lesions and distinguishing benign masses from malignant ones. Morphological features derived from CT images can serve as imaging biomarkers to stratify benign and malignant lesions. Analyses based on multivariate predictive models have confirmed that morphological parameters substantially boost diagnostic accuracy relative to conventional radiomic signatures, effectively overcoming the limitations of traditional imaging tools for differentiating benign and malignant tumors (26).

Compared with CT alone, ML and DL can optimize workflows covering image acquisition, contrast agent delivery and radiation dose. A deep learning generative adversarial network (GAN) model reduced the required volume of CT contrast agent by 50% while maintaining diagnostic consistency of image pathological features, thereby achieving optimized contrast administration (27). In contrast to standalone MRI, ML and ANNs demonstrate outstanding performance in nuclear magnetic resonance(NMR) data processing and structural prediction. Deep learning has facilitated the development of innovative NMR experimental schemes that were previously impractical, broadening the application scope and potential of biomolecular NMR spectroscopy. Such technological breakthroughs are anticipated to foster multi-layered innovations in structural biology and expedite the whole pipeline of drug discovery (28).

AI has been widely adopted in the diagnosis and treatment of various diseases. In the field of GC, AI is primarily applied to molecular bioinformatic analysis, endoscopic identification of Helicobacter pylori infection, and recognition of chronic atrophic gastritis, early GC, tumor invasion depth and pathological lesions. AI can also construct predictive models to evaluate lymph node metastasis, therapeutic response to drugs and patient prognosis. Furthermore, AI can also be utilized for surgical training, technical evaluation and intraoperative navigation (2). The application of DL models based on CT images assists radiologists in evaluating lymph node metastasis, serosal invasion and peritoneal dissemination in GC (29–31). Such models help clinicians design preoperative treatment plans and reduce unnecessary surgical interventions and associated complications. Applications of AI in the early detection of oral cancer have confirmed that DL outperforms traditional ML algorithms in diagnostic precision (32). For the challenging diagnosis of epilepsy, ML and AI play a pivotal role in interpreting electroencephalography, neuroimaging and other multimodal data (33). Regarding ultra-high-resolution coronary computed tomography angiography (UHR CCTA), a novel DL-based tool with automated case preparation functions enables quantitative analysis of coronary plaques from UHR CCTA datasets. This algorithm achieves strong robustness and excellent reproducibility, delivering anatomically consistent outputs without manual correction (34). Collectively, the integration of AI and medical imaging demonstrates promising prospects for clinical translation.

#### 4.5.3 AI and gastroscopy

Gastroscopy is the first-line examination for identifying GC and acquiring pathological tissue specimens, and pathological biopsy constitutes the gold standard for confirmed GC diagnosis. Studies comparing the diagnostic efficacy of gastric filling ultrasonography, gastroscopy, and their combined application in detecting GC have validated that gastroscopy delivers high sensitivity and specificity (35).

AI can assist endoscopic diagnosis, lower missed diagnosis rates and elevate the detection rate of upper gastrointestinal lesions (36). AI-augmented endoscopy, magnifying endoscopy and endoscopic ultrasonography significantly boost tumor detection rates (37) and reduce unnecessary biopsy procedures (38). For instance, hereditary diffuse gastric cancer (HDGC) is generally triggered by pathogenic variants in the CDH1 gene (Cadherin 1 Pathogenic Variant, CDH1-PV). Carriers face a 40%–60% lifetime risk of signet ring cell carcinoma (SRCC). Pale mucosal areas under endoscopy represent the most common lesion site of SRCC, yet such lesions are challenging to identify. A user-friendly decision tree (DT)-based diagnostic rule is expected to improve diagnostic performance for this condition. By integrating specific features of pale mucosal regions, this rule enables high-precision prediction of SRCC in HDGC and provides evidence for determining the timing of prophylactic total gastrectomy (39). In addition, AI can evaluate the adequacy of bowel preparation. Convolutional neural networks (CNNs) trained against standardized bowel preparation scoring systems such as the Boston Bowel Preparation Scale (BBPS) achieve an accuracy of up to 89.58% within 5 to 11 seconds, thereby shortening the preparation time prior to endoscopic procedures (40).

Furthermore, AI achieves high sensitivity and specificity in the endoscopic diagnosis of Helicobacter pylori (Hp) infection (41). As a gram-negative bacterium colonizing the gastric mucosa, Hp serves as a critical risk factor for GC development (42). Common clinical modalities for Hp infection detection include the urea breath test, stool antigen assay, and biopsy-based endoscopic examination (43), among which the ¹³C urea breath test is the most widely applied. The endoscopic ¹³C-urea breath test exhibits superior efficacy over the standard urea breath test for identifying Hp infection in patients after partial gastrectomy (44). Notably, AI algorithms, particularly DL models trained on image-enhanced endoscopic techniques such as laser coherent imaging (LCI) and blue laser imaging (BLI), can fully leverage the enhanced mucosal visualization and color contrast delivered by LCI and BLI. Such models precisely detect subtle mucosal alterations linked to Hp infection, post-eradication mucosal changes, and precancerous lesions including atrophic gastritis and intestinal metaplasia, substantially boosting endoscopic evaluation and detection performance for Hp infection (15). In particular, the LCI-CAD system achieves diagnostic accuracy comparable to experienced endoscopists when processing LCI images (45).

Compared with endoscopists, DL-based computer-aided diagnosis systems are not constrained by individual endoscopists’ clinical experience. They improve diagnostic accuracy for endoscopists across all experience levels, lower rates of misdiagnosis and missed lesions, and deliver superior diagnostic performance (46). Lesions originating from non-neoplastic epithelial and subepithelial tissues remain difficult to detect even with image-enhanced endoscopy (47). AI models achieve higher diagnostic accuracy than general endoscopists and boost diagnostic performance among non-specialist practitioners (48). Explainable AI builds clinicians’ confidence in AI; however, it may also cause excessive reliance on inaccurate AI recommendations, which impairs human–machine collaborative performance — one of the most commonly evaluated metrics in clinical AI studies (49). Taken together, AI serves as an indispensable auxiliary tool, yet human–machine collaboration generates better diagnostic outcomes and represents a substantial potential for future clinical practice.

#### 4.5.4 AI and radiomics

Radiomics provides a non-invasive approach for quantifying tumor heterogeneity from medical images and offers valuable insights for disease diagnosis and prognostic evaluation (50). Delta radiomics has shown promising value in therapeutic response prediction and prognostic assessment, although the overall quality of current relevant studies remains suboptimal (51). The integration of DL and radiomics facilitates non-invasive diagnosis and therapeutic decision-making for GC (52). Such hybrid models can reliably predict neoadjuvant chemotherapy response and survival outcomes of GC patients, with optimal predictive performance obtained when combined with clinical characteristic variables (53).

Relevant literature records that neural network keywords emerged in studies as early as 1993. Classic neural network architectures cover ANNs, CNNs, and recurrent neural networks (RNNs) (54). With the rapid development of AI, DL-driven CNN models exhibit excellent performance in boosting GC detection rates and forecasting tumor invasion depth (51). In addition, pathological research utilizing CNN feature extraction from histological slides of gastrointestinal tumors supports precise tumor stratification and personalized prognostic prediction (55).

#### 4.5.5 AI and multi-omics

ML enables GC diagnosis and prognostic evaluation using pathological slides, blood samples and diverse biological specimens, yet additional validations are needed to promote its clinical translation (56). DL analysis of gastrointestinal pathological sections (57) improves the accuracy of pathological subtyping and biomarker evaluation (58). Moreover, DL applied to tumor cytology (59) boosts the diagnostic sensitivity of GC and supports individualized treatment (60). Immunotherapy and the tumor microenvironment also exert vital effects on the development of precision therapy for GC (61).

Liquid biopsy has been adopted for the early screening of gastrointestinal malignancies. Multi-omics and ML-driven liquid biopsy techniques are expected to achieve precise early detection of gastrointestinal malignancies (62). Exosomes can be applied for diagnosis and disease monitoring, and also serve as potential therapeutic targets (63). Exosomal miRNAs and proteins are potential diagnostic and prognostic biomarkers (64) for dynamic surveillance and precision treatment of GC (65, 66). The combination of AI and ctDNA enhances predictive performance for tumor recurrence and patient survival (67). Accurate evaluation of PD-L1 expression is critical for therapeutic decision-making in esophageal cancer, esophagogastric junction cancer and GC (68). Meanwhile, AI improves the consistency and efficiency of detecting biomarkers such as PD-L1 and HER2 (69). Collectively, the combination of AI and liquid biopsy demonstrates a favorable trajectory for future development for the early screening of GC.

The tumor microenvironment is critical for tumor progression, and stromal cells and microenvironment remodeling participate in the initiation and progression of GC (70). Exosomes derived from GC stem cells alter gene expression and cytokine secretion in CD8+ T cells, thereby establishing an immunosuppressive microenvironment (71). Molecular and microenvironmental factors jointly drive the emergence of therapeutic resistance (72). Combined regimens consisting of anti-angiogenic therapy, radiotherapy and targeted therapy can reverse immunotherapy resistance (73).

In summary, AI improves the consistency of pathological diagnosis, the accuracy of pathological subtyping, and the capacity for prognostic prediction (74). Nevertheless, data standardization, regulatory approval and cost-effectiveness remain major obstacles to clinical translation (75). ML-driven analysis of tumor microenvironment multi-omics data from gastroesophageal adenocarcinoma supports tumor subtyping and prognostic modeling (76). The integration of AI and multi-omics streamlines GC diagnosis and prognostic assessment, and facilitates individualized treatment planning.

#### 4.5.6 AI and multimodal imaging

Recent technological advances have driven the development of multimodal generative systems. For instance, PathChat, powered by multimodal large language models (MLLMs), can process visual and textual inputs simultaneously (77). Medical large language models assist in clinical decision-making, knowledge integration and risk stratification (78). Chen et al. developed a real-time perigastric blood vessel recognition model (PGBVRM) for laparoscopic gastrectomy. Their study demonstrated that automated detection of exposed vessels allows accurate identification of abnormal blood vessels and reduces the risk of vascular injury (79). Simulation training based on augmented reality (AR) and virtual reality (VR) has been proven to enhance visuospatial cognition and psychomotor skills (80). Such tools enable preoperative surgical simulation for clinicians, boost operative proficiency and improve surgical success rates, representing a promising research on AI applications in GC treatment.

#### 4.5.7 AI and precision medicine

The future of GC treatment lies mainly in precision medicine, continuous advances in immunotherapy, novel early detection modalities, and multidisciplinary comprehensive diagnosis and treatment regimens (22). AI has gradually evolved from a simple image analysis tool into an intelligent decision-making platform integrating endoscopic, radiological, pathological and multi-omics data (81). Relevant analytical studies have demonstrated that in the precision medicine era, AI, big data and molecular subtyping facilitate individualized surgical decision-making for GC (82). AI runs through the entire workflow of GC management, including molecular profiling, endoscopic diagnosis, pathological identification, prognostic prediction and intraoperative guidance (83). Advances in GC perioperative management, multimodal therapy, minimally invasive surgery and standardized surgical procedures mirror the evolving landscape of combined therapy under the precision medicine framework (84). Furthermore, AI-assisted assessment standardizes diagnostic workflows and expands access to high-quality risk evaluation in resource-limited regions, supporting early intervention and personalized screening protocols (41). The combination of AI, multi-omics and digital healthcare is likely to represent the core directions of future clinical development.

## 5 Results

Overall, this bibliometric analysis provided a comprehensive overview of the research status and evolutionary trends of AI in GC research from 1993 to 2026. AI technologies such as DL, CNNs and radiomics have emerged as key research hotspots and cutting-edge approaches to assist endoscopic diagnosis, pathological diagnosis and immunotherapy for GC. Developing clinical AI decision-making systems capable of real-time endoscopic and pathological diagnosis, as well as comprehensive GC diagnosis integrated with genomics and proteomics, will be the core focus of future research. Nevertheless, AI will not completely replace clinicians in future clinical practice. The integration of human expertise and AI can alleviate the shortage of medical resources, professional endoscopists and pathologists in resource-limited regions and medical institutions, thereby facilitating groundbreaking advances in the diagnosis and treatment of GC.

Although AI techniques have exhibited considerable application potential in GC diagnosis, imaging analysis, risk prediction, and therapeutic decision-making, their further clinical translation still faces multiple challenges. First, most existing AI models are trained on small-sample single-center datasets, resulting in insufficient generalization capacity and poor cross-center applicability. Second, the standardization of imaging, pathological and multi-omics data is inadequate, and the mechanisms for multimodal data fusion remain immature. Third, most deep learning models lack interpretability, which undermines clinicians’ confidence and hinders their widespread clinical adoption. Fourth, existing studies mainly concentrate on model development and retrospective validation, while large-scale prospective multicenter clinical trials to provide robust clinical evidence are scarce. Fifth, although large models and multimodal intelligent systems exhibit remarkable potential, they face numerous challenges regarding data security, privacy protection, ethical guidelines and clinical translation. Accordingly, future research should prioritize multicenter data sharing and the standardization of data protocols, advance clinical validation and translational application of multimodal AI models, and ultimately achieve a transformative shift in GC care from “auxiliary lesion detection” to “intelligent clinical decision-making”.

This bibliometric study presented several limitations that warrant further improvement in future investigations. First, although supplementary analyses were conducted based on the PubMed database, the core literature retrieval was mainly performed using the WoSCC. Reliance on a single database led to incomplete literature coverage and might introduce potential selection bias. Second, non-English publications were excluded in this study, which resulted in insufficient representation of research outcomes from non-English-speaking regions. Third, conference proceedings, book chapters, editorials, and other non-original records were excluded to guarantee methodological consistency and standardized literature inclusion. However, this exclusion approach might underestimate emerging evidence and preliminary exploratory progress in this field. Fourth, data collection was completed in June 2026, and full publications of 2026 were not completely retrieved, which might subtly affect the interpretation of the latest annual research trends. Future studies should adopt multidatabase validation based on Scopus, Embase and other databases to supplement and verify the current findings, so as to further enhance the transparency and reliability of the bibliometric results.
