# Supplementary material for: Artificial intelligence in gastric cancer research: a bibliometric and visualized analysis from 1993 to 2026
Source: Front Oncol. 2026 Sep 14;16:1926967. doi: 10.3389/fonc.2026.1926967 (PMC13617442; doi:10.3389/fonc.2026.1926967)
Supplement: Supplementary file 1 [file Table1.docx]

Figure 1 Collaboration visualization map of institutions.

Figure 2 Collaboration visualization map of country/region.
